# Influence of Factors Altering Gastric Microbiota on Bariatric Surgery Metabolic Outcomes

**DOI:** 10.1128/Spectrum.00535-21

**Published:** 2021-11-17

**Authors:** Carolina Gutiérrez-Repiso, Isabel Moreno-Indias, Gracia M. Martín-Núñez, Ailec Ho-Plagaro, Luis Ocaña-Wilhelmi, Diego Fernández García, Montserrat Gonzalo Marín, Francisco J. Moreno-Ruiz, Eduardo García-Fuentes, Francisco J. Tinahones

**Affiliations:** a Unidad de Gestión Clínica de Endocrinología y Nutrición del Hospital Virgen de la Victoria, Instituto de Investigación Biomédica de Málaga (IBIMA), Málaga, Spain; b Centro de Investigación Biomédica en Red de Fisiopatología de la Obesidad y la Nutrición (CIBERobn), Instituto de Salud Carlos III, Madrid, Spain; c Unidad de Gestión Clínica de Aparato Digestivo, Hospital Universitario Virgen de la Victoria, Instituto de Investigación Biomédica de Málaga-IBIMA, Málaga, Spain; d Unidad de Gestión Clínica de Cirugía General, Digestiva y Trasplantes, Hospital Universitario Virgen de la Victoria, Departamento de Especialidades Quirúrgicas, Bioquímica e Inmunología, Universidad de Málaga, Málaga, Spain; e Unidad de Gestión Clínica de Endocrinología y Nutrición, Hospital Regional Universitario de Málaga, Instituto de Investigación Biomédica de Málaga (IBIMA), Málaga, Spain; f Unidad de Gestión Clínica de Cirugía General, Digestiva y Trasplantes, Hospital Regional Universitario de Málaga, Málaga, Spain; g Departamento de Medicina y Dermatología, Universidad de Málaga, Málaga, Spain; Lerner Research Institute

**Keywords:** microbiota, stomach, obesity, *Helicobacter pylori*, proton pump inhibitors, sleeve gastrectomy

## Abstract

Little is known about the influence of gastric microbiota on host metabolism, even though the stomach plays an important role in the production of hormones involved in body weight regulation and glucose homeostasis. Proton pump inhibitors (PPIs) and Helicobacter pylori alter gut microbiota, but their impact on gastric microbiota in patients with obesity and the influence of these factors on the metabolic response to bariatric surgery is not fully understood. Forty-one subjects with morbid obesity who underwent sleeve gastrectomy were included in this study. The H. pylori group was established by the detection of H. pylori using a sequencing-based method (*n* = 16). Individuals in whom H. pylori was not detected were classified according to PPI treatment. Gastric biopsy specimens were obtained during surgery and were analyzed by a high-throughput-sequencing method. Patients were evaluated at baseline and 3, 6, and 12 months after surgery. β-Diversity measures were able to cluster patients according to their gastric mucosa-associated microbiota composition. H. pylori and PPI treatment are presented as two important factors for gastric mucosa-associated microbiota. H. pylori reduced diversity, while PPIs altered β-diversity. Both factors induced changes in the gastric mucosa-associated microbiota composition and its predicted functions. PPI users showed lower percentages of change in the body mass index (BMI) in the short term after surgery, while the H. pylori group showed higher glucose levels and lower percentages of reduction in body weight/BMI 1 year after surgery. PPIs and H. pylori colonization could modify the gastric mucosa-associated microbiota, altering its diversity, composition, and predicted functionality. These factors may have a role in the metabolic evolution of patients undergoing bariatric surgery.

**IMPORTANCE** The gut microbiota has been shown to have an impact on host metabolism. In the stomach, factors like proton pump inhibitor treatment and Helicobacter pylori haven been suggested to alter gut microbiota; however, the influence of these factors on the metabolic response to bariatric surgery has not been fully studied. In this study, we highlight the impact of these factors on the gastric microbiota composition. Moreover, proton pump inhibitor treatment and the presence of Helicobacter pylori could have an influence on bariatric surgery outcomes, mainly on body weight loss and glucose homeostasis. Deciphering the relationship between gastric hormones and gastric microbiota and their contributions to bariatric surgery outcomes paves the way to develop gut manipulation strategies to improve the metabolic success of bariatric surgery.

## INTRODUCTION

In the last few years, there has been a boost in the number of studies evaluating the implications of gut microbiota in health and disease. The role of gut microbiota in host metabolism has been extensively probed, being shown to be involved in energy storage and body weight regulation (1). However, many of these studies have been made using fecal samples, which are representative of the large intestine microbiota. Although these studies have shed light on the composition and functionality of the gut microbiota, the number of studies evaluating the microbiota of the upper gastrointestinal tract is limited, especially for gastric microbiota.

Most of the studies evaluating gastric microbiota have been made under conditions related to the organ, such as gastric ulcer, dyspepsia, gastritis, gastroesophageal reflux disease, or Helicobacter pylori infection. However, the gastric microbiota in patients with other dysfunctions, and especially metabolic disorders like obesity, has hardly been studied (2). It should be taken into consideration that in the stomach, and especially in the gastric fundus, hormones involved in body weight regulation and glucose homeostasis are produced (3). However, few studies have been made using gastric biopsy specimens (4–6), and the microbiota of the gastric fundus is yet to be analyzed.

In animal models, a high-fat diet has been shown to induce dysbiosis in the gastric microbiota that could have a role in the development of metabolic diseases (7). But many other factors have been suggested to have an impact on gastric microbiota (2), two of which are H. pylori infection and proton pump inhibitor (PPI) treatments, which modify the gastric pH and may contribute to altering the gastric microbiota.

H. pylori has been shown to be involved in the development of diseases like gastritis, peptic ulcer disease, or gastric cancer (8). H. pylori infection has been suggested to alter gastric microbiota (9, 10), but in many cases, H. pylori infection is asymptomatic and affected individuals do not seek medical attention, so the information about the impact of H. pylori colonization on the gastric microbiota in these undiagnosed individuals is limited.

PPIs are widely used for the treatment of acid-related gastroduodenal diseases. PPIs inhibit gastric acid secretion, increasing the pH in the stomach, which can alter the gut microenvironment. Indeed, PPI use could modify the fecal microbiota composition (11, 12), but information about the effect of PPIs on gastric microbiota is limited.

A matter of debate is whether H. pylori colonization could be implicated in postoperative complications like perforations and ulcers after bariatric surgery (13, 14). However, there is no information about metabolic outcomes after bariatric surgery. Previously, we have shown that H. pylori eradication may have an impact on glucose and lipid homeostasis (15, 16), but it is not clear whether H. pylori colonization could alter the metabolic response to bariatric surgery (17). Moreover, there is no information about the influence of PPI use on bariatric surgery metabolic outcomes.

The aims of the study were to (i) investigate the composition of gastric mucosa-associated microbiota in patients with obesity, (ii) investigate the impacts of PPIs and H. pylori on the composition and function of gastric mucosa-associated microbiota in these patients, and (iii) investigate the influence of these factors on the metabolic response to bariatric surgery.

## RESULTS

### Diversity in gastric mucosa-associated microbiota.

**FIG 1:**
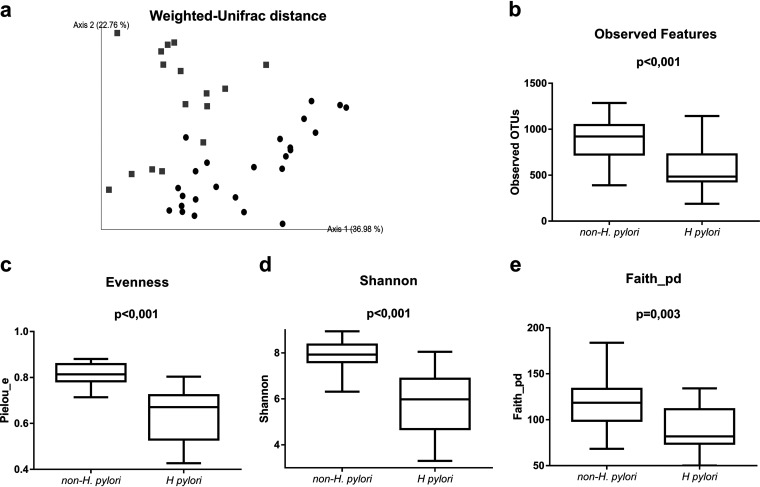
Diversity indexes of subjects classified according to the presence or absence of H. pylori, detected by a high-throughput sequencing method. (a) Weighted UniFrac distances. Gray squares, H. pylori; black circles, non-H. pylori. (b) Observed features. (c) Pielou evenness index. (d) Shannon index. (e) Faith phylogenetic diversity index.

A principal-coordinate analysis plot of weighted UniFrac distances was used to visualize complex relationships within the microbial communities. This β-diversity analysis (weighted UniFrac distance) allowed two different clusters to be distinguished according to H. pylori presence (permutational multivariate analysis of variance [PERMANOVA], pseudo-*F*, 10.93; *P* = 0.001) (Fig. 1a), as well as the unweighted UniFrac distance (PERMANOVA, pseudo-*F*, 1.87, *P* = 0.041) (data not shown). A further α-diversity analysis of richness and evenness indexes also showed significant differences depending on H. pylori presence (Fig. 1b to e).

**FIG 2:**
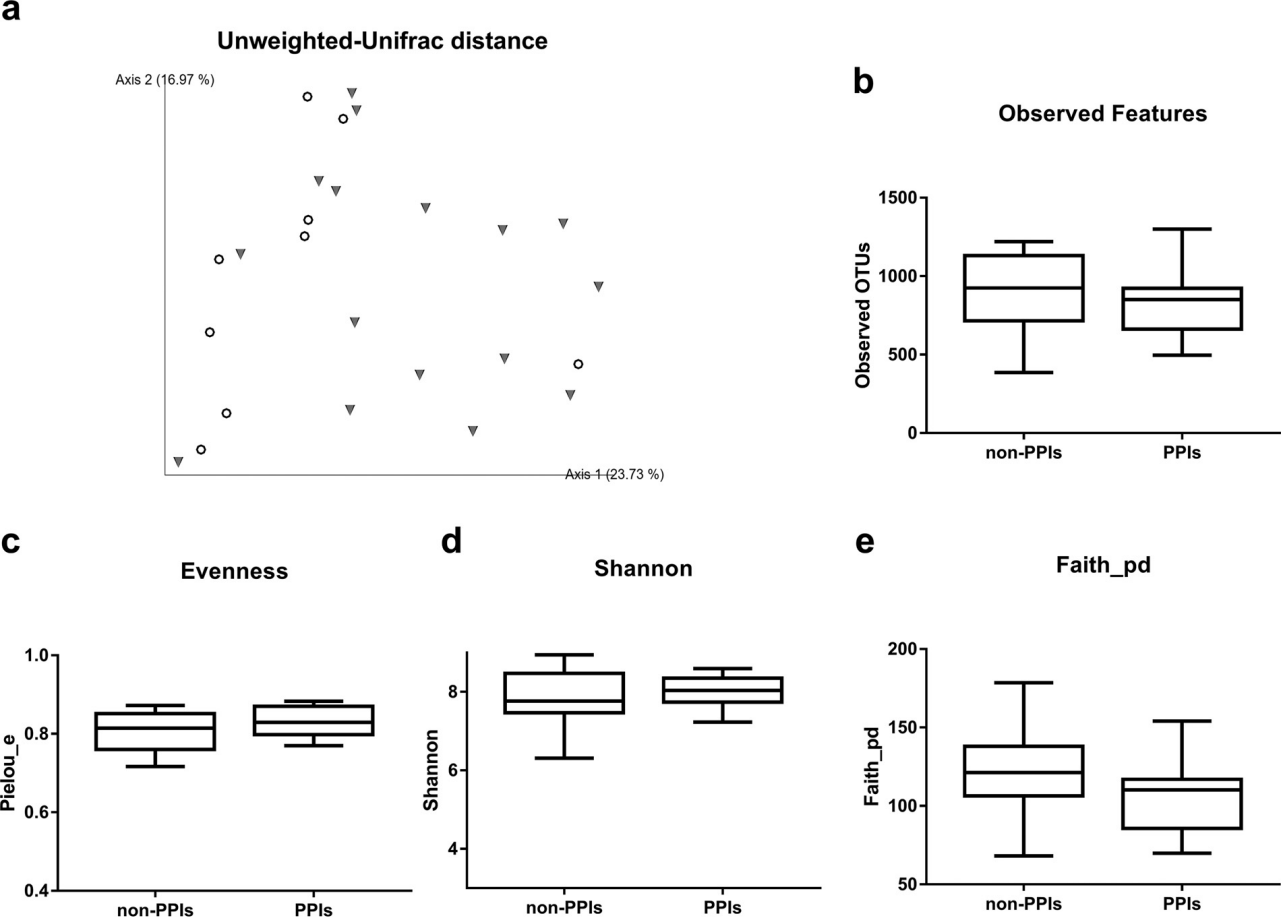
Diversity indexes of non-H. pylori subjects classified according to PPI use. (a) Unweighted UniFrac distance. Inverted triangles, non-PPI users; white circles, PPI users. (b) Observed features. (c) Pielou evenness index. (d) Shannon index. (e) Faith phylogenetic diversity index.

Deepening into the non-H. pylori group and following the same strategy, the unweighted UniFrac distance measure showed different clusters according to PPI use (PERMANOVA, pseudo-*F*, 1.79; *P* = 0.047) (Fig. 2a). However, no statistically significant differences according to α-diversity were found between these two clusters (Fig. 2b to e).

### Anthropometric and biochemical characteristics.

Based on the differences detected in microbial diversity analysis, subjects were classified into three groups: PPI users, non-PPI users, and the H. pylori group. These groups were used throughout the study. The main characteristics of the studied groups are represented in Table 1. At baseline, the mean age of the non-PPI users was significantly lower than the mean ages of the PPI users (*P* = 0.021) and the H. pylori group (*P* = 0.006). No differences were found in the rest of the studied variables at baseline.

**TABLE 1:** Anthropometric and biochemical characteristics of the patients included in the study at different study time points

Characteristic^*a*^	Time point	Mean value ± SD for^*b*^:		
		Non-PPI users	PPI users	H. pylori
Sex (M/F)		3/13	1/8	5/11
Age (yr)		36.87 ± 7.76	48.56 ± 10.43*	47.27 ± 9.43†
Weight (kg)	Baseline	137.12 ± 19.23	128.32 ± 24.03	128.29 ± 24.48
	3 mo	110.29 ± 17.53	105.96 ± 20.33	103.85 ± 22.19
	6 mo	99.57 ± 15.13	87.63 ± 12.98	94.11 ± 22.90
	1 yr	85.46 ± 14.06	81.28 ± 8.9	92.02 ± 18.82
BMI (kg/m^2^)	Baseline	50.06 ± 4.77	49.69 ± 7.93	48.02 ± 9.87
	3 mo	39.42 ± 4.61	39.85 ± 6.26	39.27 ± 9.29
	6 mo	35.14 ± 4.30	34.07 ± 6.25	35.94 ± 9.94
	1 yr	30.29 ± 4.23	32.19 ± 4.32	34.99 ± 8.97
Glucose (mg/dl)	Baseline	90.88 ± 13.41	94.89 ± 19.21	104.19 ± 38.49
	3 mo	75.5 ± 8.28	85.33 ± 7.23	85-67 ± 13.74
	6 mo	77.33 ± 11.60	82.00 ± 7.61	87.6 ± 12.66
	1 yr	73.00 ± 8.74	80.00 ± 9.52	87.91 ± 15.01†
Insulin (µUI/ml)	Baseline	26.45 ± 13.97	21.67 ± 15.10	23.46 ± 17.15
	3 mo	9.23 ± 5.37	9.81 ± 5.61	11.84 ± 4.88
	6 mo	9.28 ± 5.03	9.36 ± 3.22	12.32 ± 11.46
	1 yr	6.69 ± 2.18	7.19 ± 4.00	8.86 ± 6.89
HOMA-IR	Baseline	5.95 ± 3.12	5.64 ± 5.15	6.47 ± 5.78
	3 mo	1.7 ± 0.94	2.12 ± 1.36	2.47 ± 0.99
	6 mo	1.86 ± 1.32	1.86 ± 0.55	2.66 ± 2.67
	1 yr	1.21 ± 0.46	1.48 ± 0.97	1.84 ± 1.63
HbA1c (%)	Baseline	5.56 ± 0.56	5.66 ± 0.45	6.11 ± 1.11
	3 mo	5.1 ± 0.47	5.40 ± 0.21	5.40 ± 0.31
	6 mo	5.07 ± 0.51	5.37 ± 0.19	5.45 ± 0.34
	1 yr	5.092 ± 0.46	5.33 ± 0.23	5.32 ± 0.28
Cholesterol (mg/dl)	Baseline	172.38 ± 27.43	171.00 ± 34.52	183.00 ± 35.19
	3 mo	171.83 ± 28.19	173.5 ± 31.88	183.25 ± 32.14
	6 mo	166.42 ± 35.18	194.5 ± 21.76	199.4 ± 33.10
	1 yr	177.41 ± 41.56	186.83 ± 44.49	184.36 ± 32.39
Triglycerides (mg/dl)	Baseline	112.19 ± 50.48	127.33 ± 53.63	166.87 ± 109.253
	3 mo	100.17 ± 29.11	110.83 ± 50.95	122.58 ± 64.55
	6 mo	83.17 ± 20.07	91.75 ± 37.74	104.5 ± 57.16
	1 yr	65.67 ± 15.04	104.83 ± 40.99	90.73 ± 53.35
HDL-chol (mg/dl)	Baseline	43.44 ± 10.45	51.89 ± 11.53	46.25 ± 10.54
	3 mo	38.67 ± 6.38	48.17 ± 7.05	46.42 ± 11.74
	6 mo	41.25 ± 3.67	59.5 ± 11.50	50.50 ± 15.99
	1 yr	49.33 ± 9.84	61.33 ± 18.25	53.54 ± 12.94
LDL-chol (mg/dl)	Baseline	106.5 ± 25.98	93.64 ± 28.98	104.46 ± 33.13
	3 mo	113.08 ± 28.65	103.16 ± 25.46	112.31 ± 27.11
	6 mo	108.53 ± 30.51	116.65 ± 27.61	128.00 ± 30.22
	1 yr	115.00 ± 36.66	104.60 ± 33.34	112.67 ± 28.02

a BMI, body mass index; HOMA-IR, homeostasis model assessment of insulin resistance index; HbA1c, glycated hemoglobin.

b *, Kruskal-Wallis test, *P* < 0.05 between non-PPI users and PPI users; †, *P* < 0.05 between non-PPI users and H. pylori group.

### Gastric core microbiota of patients with morbid obesity.

**FIG 3:**
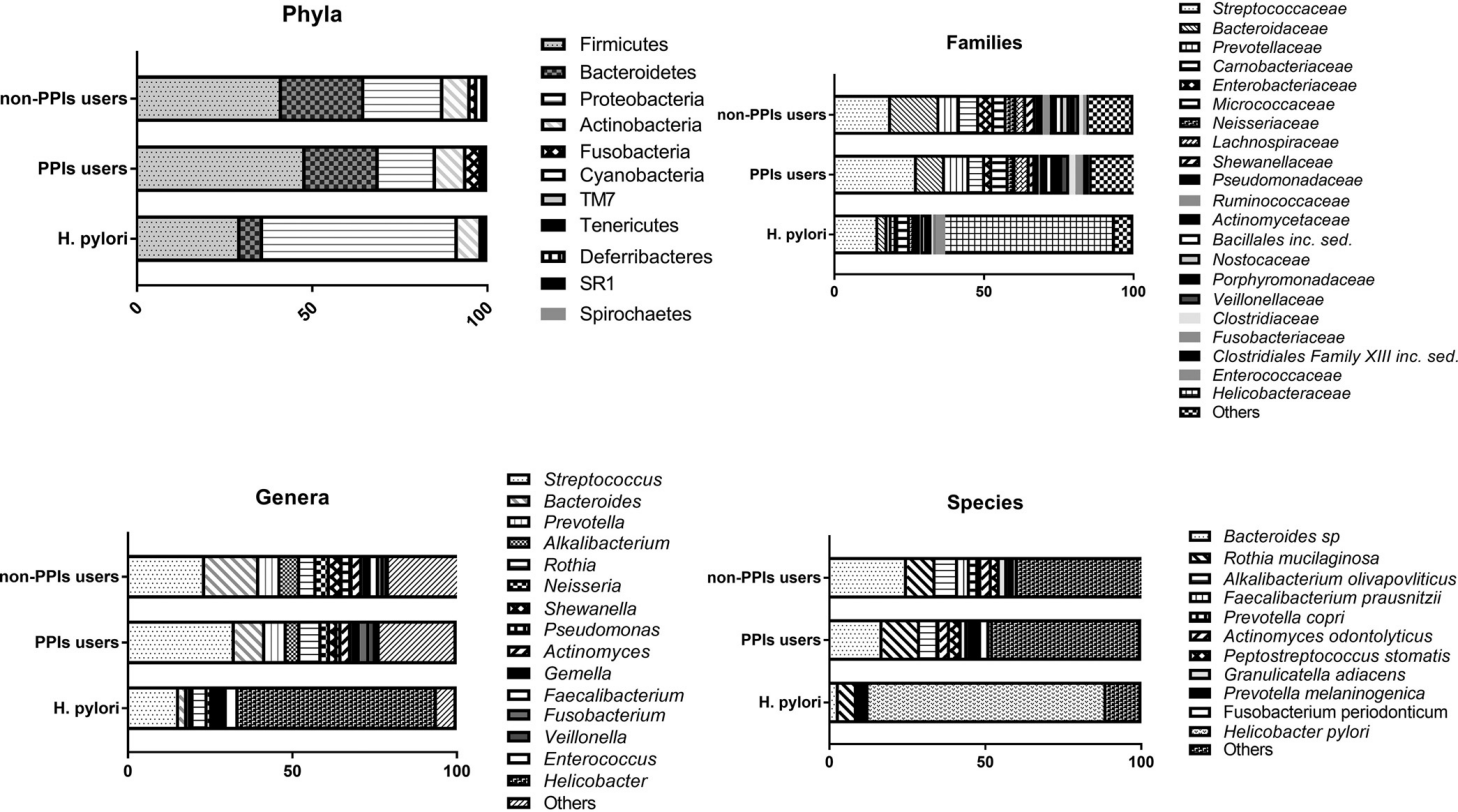
Most-highly represented taxa in gastric mucosa-associated microbiota of patients with morbid obesity, grouped into PPI users (non-H. pylori) and non-PPI users (non-H. pylori) and the H. pylori group.

The gastric mucosa-associated microbiota of all subjects included in the study are represented in Fig. S1 in the supplemental material. Focusing on the gastric mucosa-associated microbiota of those subjects without factors that alter gastric microbiota, that is, the non-PPI group, this group was mainly represented by six phyla: *Firmicutes* (39.73%), *Bacteroidetes* (22.55%), *Proteobacteria* (21.66%), *Actinobacteria* (7.45%), *Fusobacteria* (1.83%), and *Cyanobacteria* (1.78%). The predominant families were *Streptococcaceae* (18.92%), *Bacteroidaceae* (16.18%), *Prevotellaceae* (6.87%), *Carnobacteriaceae* (6.42%), *Enterobacteriaceae* (4.99%), *Micrococcaceae* (4.15%), *Neisseriaceae* (3.30%), and *Lachnospiraceae* (3.24%). At the genus level, Streptococcus was the genus most represented (23.47%), followed by *Bacteroides* (16.45%), *Prevotella* (6.44%), *Alkalibacterium* (5.96%), *Rothia* (4.97%), *Neisseria* (4.05%), *Shewanella* (3.89%), Pseudomonas (2.99%), and *Actinomyces* (2.93%). And finally, at the species level, *Bacteroides* species were the most predominant species (24.93%), followed by Rothia mucilaginosa (9.10%), Alkalibacterium olivapovliticus (7.24%), Faecalibacterium prausnitzii (3.80%), Prevotella copri (3.63%), and Actinomyces odontolyticus (3.36%) (Fig. 3).

### Impacts of PPI use and H. pylori on the gastric mucosa-associated microbiota core.

In 100% of the studied samples, the families *Bacteroidaceae*, *Streptococcaceae*, *Carnobacteriaceae*, and *Micrococcaceae* were detected, as well as members of the *Bacteroides* and Streptococcus genera and *Bacteroides* species (Fig. S2a).

At the family level, *Prevotellaceae*, *Lachnospiraceae*, *Veillonellaceae*, *Shewanellaceae*, *Clostridiaceae*, *Lactobacillaceae*, *Coriobacteriaceae*, *Campylobacteraceae*, and *Enterobacteriaceae* were found in 100% of samples in both the PPI and the non-PPI group, while *Porphyromonadaceae*, *Fusobacteriaceae*, *Peptostreptococcaceae*, and *Flavobacteriaceae* were found in all non-PPI users and *Actinomycetaceae*, *Neisseriaceae*, *Bacillales incertae sedis*, *Erysipelotrichaceae*, and *Clostridiales* Family XI were found in all PPI users (Fig. S2a).

At the genus level, *Alkalibacterium* and *Shewanella* were identified in 100% of the PPI and non-PPI groups, while *Fusobacterium* and *Eubacterium* were detected in non-PPI users and *Actinomyces*, *Rothia*, *Granulicatella*, *Prevotella*, *Neisseria*, *Gemella*, *Veillonella*, *Atopobium*, *Clostridium*, and *Oribacterium* were identified in PPI users (Fig. S2b).

At the species level, only Alkalibacterium olivapovliticus was represented in 100% of the PPI and non-PPI groups, while Rothia mucilaginosa, Granulicatella adiacens, Atopobium parvulum, Oribacterium sinus, Gemella haemolysans, and Streptococcus pseudopneumoniae were identified in PPI users (Fig. S2c).

### Impacts of PPI use and H. pylori on the gastric mucosa-associated microbiota composition.

**FIG 4:**
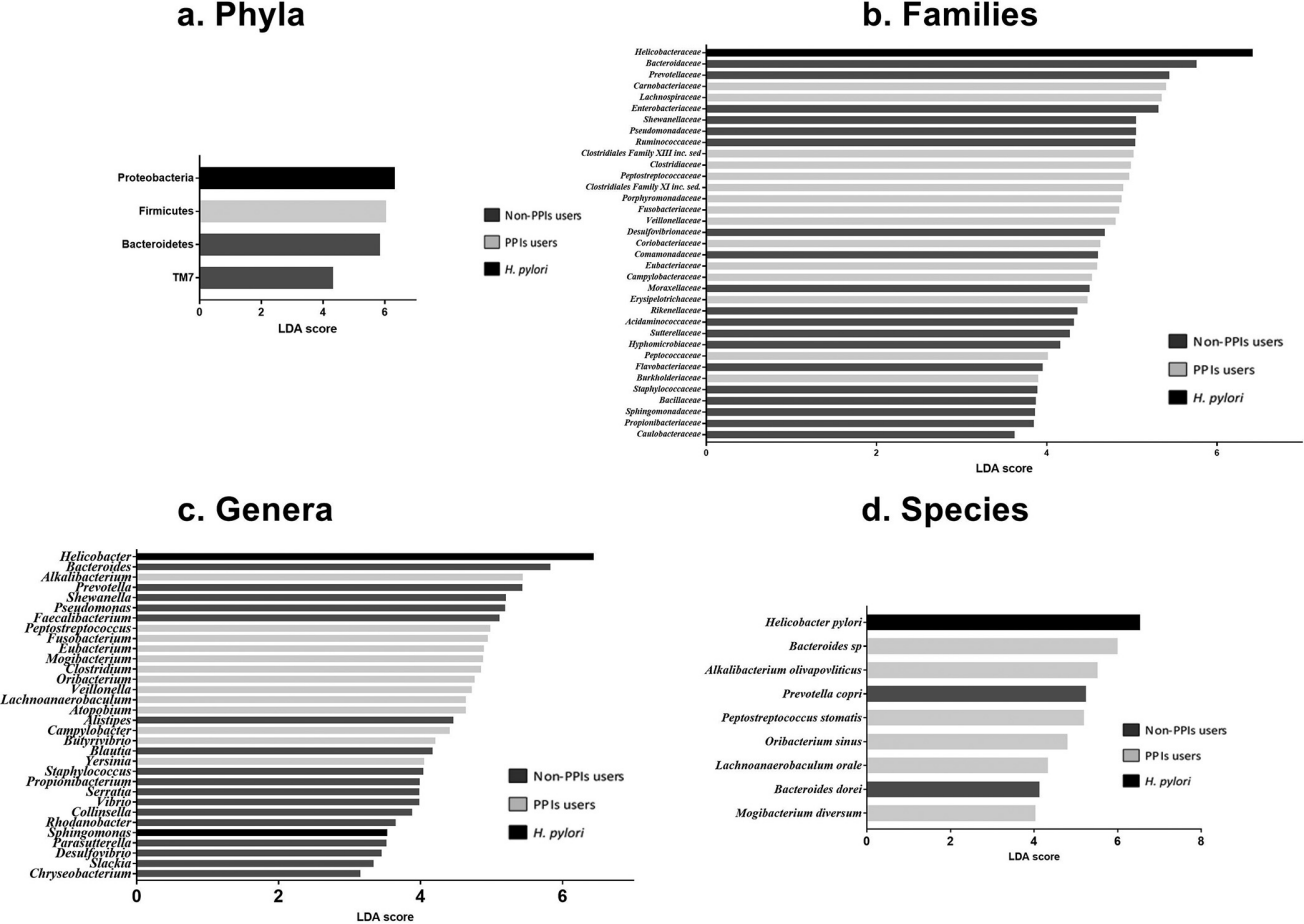
Linear discriminant analysis score of LEfSe analysis at different levels. Dark gray bars, non-PPI users; light gray bars, PPI users; black bars, H. pylori patients.

At the phylum level, linear discriminant analysis (LDA) effect size (LEfSe) analysis revealed that the non-PPI group was enriched in *Bacteroidetes* and TM7, while *Firmicutes* was predominant in the PPI group and the H. pylori group was enriched in *Proteobacteria* (LDA > 2; false discovery rate [FDR]-adjusted *P* value, <0.05) (Fig. 4a).

At the family level, the non-PPI group was enriched in *Bacteroidaceae*, *Prevotellaceae*, *Enterobacteriaceae*, *Shewanellaceae*, *Pseudomonadaceae*, *Ruminococcaceae*, *Desulfovibrionaceae*, *Comamonadaceae*, *Moraxellaceae*, *Rikenellaceae*, *Acidaminococcaceae*, *Sutterellaceae*, *Hyphomicrobiaceae*, *Flavobacteriaceae*, *Staphylococcaceae*, *Bacillaceae*, *Sphingomonadaceae*, *Propionibacteriaceae*, and *Caulobacteraceae*. The PPI group was enriched in *Carnobacteriaceae*, *Lachnospiraceae*, *Clostridiales* Family XIII *incertae sedis*, *Clostridiaceae*, *Peptostreptococcaceae*, *Clostridiales* Family XI *incertae sedis*, *Porphyromonadaceae*, *Fusobacteriaceae*, *Veillonellaceae*, *Coriobacteriaceae*, *Eubacteriaceae*, *Campylobacteraceae*, *Erysipelotrichaceae*, *Peptococcaceae*, and *Burkholderiaceae*, while the H. pylori group was characterized by an enrichment in *Helicobacteraceae* (LDA > 2; FDR-adjusted *P* value, <0.05) (Fig. 4b).

At the genus level, the non-PPI group was enriched in *Bacteroides*, *Prevotella*, *Shewanella*, Pseudomonas, *Faecalibacterium*, *Alistipes*, *Blautia*, Staphylococcus, *Propionibacterium*, *Serratia*, *Vibrio*, *Collinsella*, *Rhodanobacter*, *Parasutterella*, *Desulfovibrio*, *Slackia*, and *Chryseobacterium*. The PPI group was enriched in *Alkalibacterium*, *Peptostreptococcus*, *Fusobacterium*, *Eubacterium*, *Mogibacterium*, *Clostridium*, *Odoribacter*, *Veillonella*, *Lachnoanaerobaculum*, *Atopobium*, Campylobacter, *Butyrivibrio*, and *Yersinia*, while in the H. pylori group, enrichment in *Helicobacter* and *Sphingomonas* was found (LDA > 2; FDR-adjusted *P* value, <0.05) (Fig. 4c).

At the species level, the non-PPI group was enriched in Prevotella copri and Bacteroides dorei, while the PPI group was enriched in *Bacteroides* species, Alkalibacterium olivapovliticus, Peptostreptococcus stomatis, Oribacterium sinus, Lachnoanaerobaculum orale, and Mogibacterium diversum. As expected, H. pylori was the predominant species in the H. pylori group (LDA > 2; FDR-adjusted *P* value, <0.05) (Fig. 4d), with abundances that ranged from 5.49% to 56.13%.

### Predicted metabolic pathways in gastric mucosa-associated microbiota.

MetaCyc pathway analysis was performed to gain a better understanding of the role of gastric microbiota in each of the studied groups. This analysis estimated that the H. pylori group was enriched in pathways involved in the biosynthesis of tetrapyrroles, proteinogenic amino acids (l-lysine, l-isoleucine, l-threonine, and l-tryptophan), heme b, quinol and quinone, cell wall, phospholipids, nucleosides, nucleotides, and sugars and also in pathways involved in the generation of precursors of metabolites and energy (Table S1), while a decrease was shown in predicted pathways involved in the degradation of aromatic compounds, l-arginine, polysaccharides (glycan and glycogen), and sugar acids (d-galactarate, d-galacturonate, and d-glucarate) and also in pathways involved in the fermentation of pyruvate and fermentation to short-chain fatty acids and the biosynthesis of proteinogenic amino acids (l-arginine, l-histidine, and l-arginine), fatty acids (palmitate, stearate, oleate, and palmitoleate), thiamine, and sugar nucleotides (Table S1).

Comparing PPI users with non-PPI users, we found that there was an enrichment in sugar degradation in PPI users, while the non-PPI group was enriched in pathways involving aromatic compound degradation (benzoyl-CoA, protocatechuate, and vanillin) and nucleoside and nucleotide degradation, among others (Fig. S3).

### Impacts of PPI use and H. pylori on the evolution of metabolic response to bariatric surgery.

Glucose levels at the 1-year follow-up after bariatric surgery were significantly higher in the H. pylori group than in non-PPI users (*P* = 0.042). There were no statistically significant differences between groups in the rest of the variables at the different study time points (Table 1).

**FIG 5:**
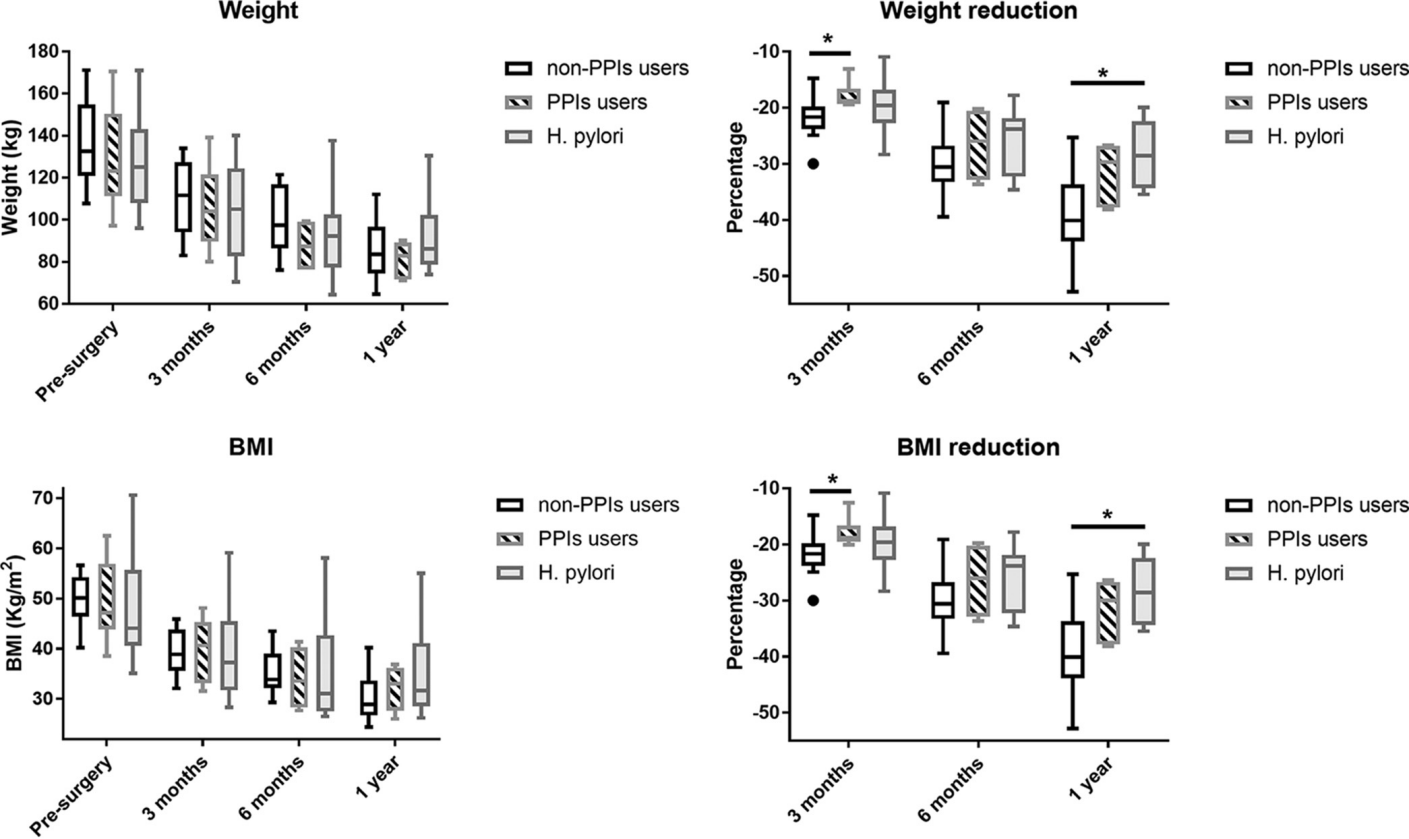
Weight/BMI trajectories and their percentages of reduction during follow-up. *, *P* < 0.05 (Kruskal-Wallis test adjusted by the Bonferroni correction for multiple tests).

If variables are analyzed as percentages of change throughout the follow-up, the changes in weight and in body mass index (BMI) at 3 months were significantly higher in the non-PPI than in the PPI group (−21.81 ± 3.67 [mean ± standard deviation] versus −17.84 ± 2.42 [*P* = 0.028] and −21.81 ± 3.66 versus 17.89 ± 2.70 [*P* = 0.042], respectively). The percentages of changes of weight and BMI at the 1-year follow-up were significantly higher in non-PPI users than in the H. pylori group (−38.96 ± 7.48 versus −28.38 ± 5.63 [*P* = 0.005] and −38.95 ± 7.49 versus −28.39 ± 5.63 [*P* = 0.005]). The weight/BMI trajectories and their percentages of reduction during the follow-up are shown in Fig. 5. There were no statistically significant differences between groups in the rest of the variables at the different study time points (Table S2).

## DISCUSSION

Gastric cancer, H. pylori infection, dyspepsia, and gastritis, among other diseases, have drawn the attention of gastric microbiota studies. Even though the gut microbiota has been shown to have an impact on host metabolism and the stomach has an important role in the production of hormones involved in body weight regulation and glucose homeostasis, knowledge about the relation between gastric microbiota and obesity is scarce. The present study describes the gastric mucosa-associated microbiota in patients with morbid obesity, and what is more important, we have shown the utility of the gastric mucosa-associated microbiota to find similar patterns among patients. Indeed, we showed the impact of commonly used medications, such as PPIs, and alterations, such as H. pylori presence, on the gastric microbiota composition and functions. On the one hand, although it produced alterations in the gastric microbiota composition, PPI treatment did not have a direct impact on its diversity; besides this, it produced a smaller BMI change in the short term after bariatric surgery. On the other hand, individuals not diagnosed with H. pylori showed lower microbiota diversity and also smaller reductions in body weight/BMI and higher glucose levels after bariatric surgery, something that deserves further investigation.

So far, there are few articles that have evaluated the gastric microbiota using biopsy specimens and high-throughput analysis for 16S rRNA sequencing approaches (18, 19), particularly in patients with morbid obesity, hindering the comparison of results between studies. In our study, as expected, the main phyla were *Firmicutes*, *Bacteroidetes*, *Proteobacteria*, *Actinobacteria*, and *Fusobacteria*, as has been previously described (18–20). Our study provides knowledge about the gastric mucosa of patients with morbid obesity. The results showed that the predominant families were *Streptococcaceae* and *Bacteroidaceae*, as well as their respective genera Streptococcus and *Bacteroides*. Streptococcus is an acid-resistant bacterium (21), while *Bacteroides* is one of the most abundant bacteria in the gastrointestinal tract and a mutualistic bacterium that is key for the normal operation of the rest of the microbiota members (22). Thus, both bacteria seem to be key for the gastric microbiota function.

The presence of a halotolerant bacterium (*Alkalibacterium olivoapovliticus*) in the human gastric microbiota should be highlighted. Previous studies have reported the presence of halophilic and halotolerant microorganisms in human microbiota (23). Moreover, a link between halophilic microbiota and obesity has been suggested (24). Further studies are warranted to determine the role of halophilic and halotolerant bacteria in the human gastrointestinal tract and its possible link with diseases like obesity.

Previously, it has been suggested that H. pylori infection can alter the gastric microbiota composition (9, 10, 25). In this regard, our results showed that H. pylori colonization had an impact on the α- and β-diversity of gastric microbiota, although others, also using a sequencing approach, did not find discrepancies in diversity between patients positive and negative for H. pylori (19). However, in the latter study, the authors detected that individuals who were H. pylori negative when tested by conventional methods could hide the presence of H. pylori that was detected with the sensitive high-throughput sequencing method, which could hide the real impact of H. pylori colonization on gastric microbiota diversity and composition (19), supporting our approach.

LEfSe analysis showed an enrichment in the *Sphingomonas* genus in the H. pylori group. *Sphingomonas* has been identified as a player in the immune response, producing glycosphingolipids that correlate with natural killer T cells (26) Moreover, the lack of *Sphingomonas* after an H. pylori eradication treatment has been related to a persistent inflammatory state (27), indicating some kind of crucial role in the gastric microbiota but at a correct amount, although further studies are warranted to elucidate the role of *Sphingomonas*.

PPI use is increasing because of their multiple indications. Previous studies have been focused on the impact of PPIs on distal gut microbiota. A recent systematic review concluded that PPIs do not affect diversity but that they are associated with taxonomic alterations in the upper and distal gut microbiota (28). However, few studies have been done using gastric biopsy specimens. These studies have pointed out that PPI-treated patients were enriched in Streptococcus (5, 6). Although *Streptococcaceae* have been found as part of the core group of the gastric microbiota, we did not find this association between Streptococcus and PPI treatment. The direct consequence of the use of PPIs is the increase in the gastric pH, which allows higher colonization of the gastric environment by oral and upper gastrointestinal tract commensals, something reflected in our study, where PPI-treated patients’ gastric microbiota showed enrichment in taxa frequently found in oral microbiota (29, 30). Our results are consistent with those of the prospective cohort study published by Amir et al. In that study, the authors showed that in gastric fluid, PPI treatment produced an enrichment in the oral microbiota members *Erysipelotrichaceae* and *Clostridiales*, while *Moraxellaceae*, *Comamonadaceae*, and *Flavobacteriaceae* decreased (31).

PPI-treated patients showed enrichment of Peptostreptococcus stomatis, a known member of the oral microbiome that has been associated with colorectal and gastric cancer (32, 33), as well as of Lachnoanaerobaculum orale and Mogibacterium diversum, both of which have been isolated in oral cavities, although there is no information about their possible role and functions in the gastrointestinal tract (34, 35). However, members of the oral microbiota have been related to a variety of systemic diseases when their abundances increase beyond the mouth (36).

Our results, based on patients with morbid obesity, showed enrichment in Prevotella copri and Bacteroides dorei. Although it has been shown to be one of the dominant species in the gastric microbiota (37), P. copri has shown contradictory associations with both benefits to the host and disease in the human gut, being related to glucose metabolism improvement, mainly through the formation of short-chain fatty acids, but also to proinflammatory status promotion and the ability to potentiate the pathogenicity of other bacteria (38). So far as we know, B. dorei has not been previously detected in the gastric microbiota. In the intestinal microbiota, there are contradictory results regarding the inflammatory or anti-inflammatory role of B. dorei (39, 40). Further studies are necessary to elucidate the functions that the microbiota carries out in the stomach.

In the H. pylori group, predicted MetaCyc pathways showed an enrichment in pathways that are the basis of cellular activities involved in physiological processes like nucleoside and nucleotide biosynthesis, phospholipid biosynthesis, and the generation of precursor metabolites and energy. Also, heme biosynthesis and the intermediates of its biosynthesis as porphyrins were shown to be enriched. Heme represents a key iron source and an essential growth factor for bacteria that contributes to their virulence (41). Previous studies have shown that heme biosynthesis is increased in H. pylori infection, and H. pylori eradication has been shown to downregulate tetrapyrroles, which are involved in heme biosynthesis (42). In fact, although this has not been observed in our patients, H. pylori infection increases the risk for low iron status (43), as degradation of lysosomal ferritin may facilitate H. pylori’s pathogenesis and contribute to the bacterial persistence in the human stomach (44). Cytochromes are proteins containing heme groups, and both quinones and cytochromes are components of the H. pylori respiratory chain (45). In this manner, our results showed that the quinol and quinone biosynthesis predicted pathway was enriched in patients with H. pylori.

H. pylori eradication prior to bariatric surgery has been suggested to avoid postoperative complications, although the clinical evidence is scarce (13, 14). However, to the best of our knowledge, there is no study that has evaluated the impact of the presence of H. pylori, not detected before bariatric surgery, on the metabolic response to surgery. Our results showed that 1 year after bariatric surgery, glucose levels were significantly higher in the H. pylori group than in non-PPI users, although we did not find differences in the percentages of change in glucose levels during the follow-up, while the percentages of weight/BMI reduction were significantly lower. In a recent study, the impact of H. pylori eradication on the metabolic response to bariatric surgery has been evaluated, showing a greater reduction in BMI at 3 months after surgery in patients treated for H. pylori (17). Previous studies have suggested that H. pylori infection may affect gastric hormones involved in appetite regulation, such as ghrelin and obestatin (46), that are mainly produced in the gastric fundus, so the relation between H. pylori infection, gastric hormones, and bariatric surgery is an issue that deserves further investigation. Indeed, the role of gastric hormones in bariatric surgery outcomes has been brought to light by a recent study that suggests a reprogramming of endocrine cell differentiation after bariatric surgery (47). Elucidating the impact of gastric microbiota in this adaptative process is an issue that will be worth further research.

Several limitations to this research must be acknowledged. Patients were not tested for H. pylori infection by conventional methods before surgery, so we cannot claim infection, only presence. and biopsy specimens were obtained from only one location in the stomach. The study only included patients who underwent sleeve gastrectomy, but Roux-en-Y gastric surgery also induces stomach rearrangements, so further studies will be necessary to elucidate the role of gastric microbiota in the outcomes of different bariatric surgery procedures.

In conclusion, in patients with morbid obesity, PPI treatment, although it did not alter microbiota diversity, had an impact on the composition and function of gastric mucosa-associated microbiota. The presence of H. pylori produced a reduction in gastric mucosa-associated microbiota diversity, and it was associated with smaller weight/BMI reductions 1 year after surgery and could have had an impact on glucose metabolism. This study remarks the importance of gut microbiota in the metabolic machinery of the host and the necessity to study other gastrointestinal sections apart from the large intestine to fully understand the role of gastrointestinal microbiota in the homeostasis of the host. Moreover, this study points out the use of the gastric mucosa-associated microbiota in particular, and gut microbiota in general, for the development of personalized medicine.

## MATERIALS AND METHODS

### Subjects.

The study was undertaken in 41 subjects with morbid obesity (BMI > 40 kg/m^2^) or with a BMI of >35 kg/m^2^ with comorbidities (the BMIs ranged from 35.1 kg/m2 to 70.6 kg/m^2^) who underwent sleeve gastrectomy from 2014 to 2018 at the Regional University Hospital and Virgen de la Victoria University Hospital, Málaga, Spain. Subjects were excluded if they had type 2 diabetes and were receiving insulin treatment or had cardiovascular disease or acute inflammatory or infectious disease. Any use of antibiotics or probiotic or prebiotic agents that could modify the microbiota in the previous 3 months were grounds for exclusion. PPI use was recorded. Patients followed a very-low-calorie diet (600 to 800 kcal) for 15 days before surgery (Optisource; Nestlé Health Care Nutrition), supplemented with proteins (1 g/kg of ideal body weight per day; ideal weight was defined by the weight corresponding to a body mass index [BMI] of 25).

All the participants included in the study gave their informed consent, and the study was reviewed and approved by the Ethics and Research Committee of the Regional University Hospital and Virgen de la Victoria University, Málaga, Spain. Samples from subjects were processed and frozen immediately after their reception in the Regional University Hospital Biobank (Andalusian Public Health System Biobank).

### Anthropometric and laboratory measurements.

Patients were followed up for 1 year after surgery. Anthropometric and laboratory measurements were recorded before surgery and 3, 6, and 12 months after surgery. Weight and height were measured according to standardized procedures, and BMI was calculated as weight (kg)/height^2^ (m^2^).

At all study time points, blood samples were collected after a 10- to 12-h fast. The serum was separated and immediately frozen at −80°C until analysis. Biochemical variables were measured in duplicate. Glucose, cholesterol, and triglycerides (Rando Laboratories, Antrium, UK) were determined by standard enzymatic methods. Insulin was determined by radioimmunoassay (DIASource ImmunoAssays SA, Nivelles, Belgium). The HOMA-IR (homeostasis model assessment of insulin resistance index) value was calculated with the following equation: HOMA-IR = fasting insulin (µIU/ml) × fasting glucose (mmol/liter)/22.5.

### Analysis of gastric mucosa-associated microbiota.

Gastric samples were obtained during the sleeve gastrectomy bariatric surgery. Samples were frozen in liquid nitrogen and maintained at −80°C until analysis. Gastric mucosa was scraped, and DNA was obtained using the PowerFecal DNA isolation kit (MoBio Laboratories, Inc., Carlsbad, CA, USA) according to the manufacturer’s instructions, with an initial enzymatic treatment consisting of a lysis buffer containing 20 mM Tris-HCl, 2 mM EDTA, 1.2% Triton X-100, 10 mg/ml lysozyme, and 100 U/ml mutanolysin.

Amplicon library preparation was performed using the Ion 16S metagenomics kit and Ion Plus fragment library kit (Thermo Fisher Scientific, Inc., Waltham, MA, USA) as previously described (48).

Emulsion PCR was carried out using the Ion Chef System (Thermo Fisher Scientific, Inc., Waltham, MA, USA) according to the manufacturer’s instructions. Sequencing of the amplicon libraries was carried out on an Ion 520 chip using the Ion Torrent S5 (Thermo Fisher Scientific, Inc., Waltham, MA, USA) according to the manufacturer’s instructions.

### Sequence data analysis.

Base calling and run demultiplexing were performed using Torrent Suite software version 5.4.0 (Life Technologies) with the default parameters (bead loading ≤30, key signal ≤30, and usable sequences ≤30). Data were analyzed using Quantitative Insights Into Microbial Ecology 2 (QIIME2) version 2019.1. Sequencing reads were denoised and clustered into amplicon sequence variants using DADA2 (49). Sequences were taxonomically classified against Greengenes 13_8 97% operational taxonomic unit (OTU) reference sequences. A randomly subsample at the lowest number of reads were used to evaluate alpha (Shannon’s diversity, Pielou’s evenness, Faith’s phylogenetic diversity, and observed amplicon sequence variants [ASVs]) and beta diversity (Unweighted Unifrac and Weighted Unifrac distances). Samples with fewer than 1,500 sequences were removed prior to the analysis. Features with a count sum of less than 10 across all samples and those presented only in one sample were excluded from further analysis.

ASV tables were analyzed at different taxon levels using LEfSe to test differences in abundance between groups within the MicrobiomeAnalyst webtool with the default parameters of the developer (50). The plug-in feature-table in QIIME2 was used to calculate the core microbiomes, and the visualization was performed using the Venny web tool (51).

Identification of predicted metagenome functions was performed using the Phylogenetic Investigation of Communities by Reconstruction of Unobserved States plug-in (PICRUST2). MetaCyc pathways (52) were normalized within QIIME2 and analyzed using the STAMP (Statistical Analysis of Metagenomics Profiles) software with Welch’s *t* test option (53).

### Anthropometric and biochemical statistical analysis.

Statistical software package SPSS version 22.0 (SPSS, Inc., Chicago, IL, USA) was used to study differences in anthropometric and biochemical variables between groups, using the Kruskal-Wallis test adjusted by the Bonferroni correction for multiple tests. Values were considered to be statistically significant when the *P* value was <0.05. The results are given as the mean values or percentages ± standard deviations.

### Data availability.

Raw 16S rRNA sequencing data for all samples have been deposited in the NCBI short read archive under accession number PRJNA706562.
