# Supplementary material for: Influence of Factors Altering Gastric Microbiota on Bariatric Surgery Metabolic Outcomes
Source: Microbiol Spectr. 2021 Nov 17;9(3):e00535-21. doi: 10.1128/Spectrum.00535-21 (PMC8597636; doi:10.1128/Spectrum.00535-21)
Supplement: SUPPLEMENTAL FILE 1 — Supplemental material. Download SPECTRUM00535-21_Supp_1_seq10.pdf, PDF file, 3.9 MB [file spectrum00535-21_supp_1_seq10.pdf]

Supplementary Table 1

Common name	H. pylori patients	Pathway ID	Superclass
4-hydroxyphenylacetate degradation	Decreased	3-HYDROXYPHENYLACETATE-DEGRADATION-PWY	Degradation/Utilization/Assimilation → Aromatic Compound Degradation
superpathway of L-arginine, putrescine, and 4-aminobutanoate degradation	Decreased	ARGDEG-PWY	Degradation/Utilization/Assimilation → Amino Acid Degradation → Proteinogenic Amino Acid Degradation → L-arginine Degradation
L-arginine biosynthesis I (via L-ornithine)	Decreased	ARGSYN-PWY	Biosynthesis → Amino Acid Biosynthesis → Proteinogenic Amino Acid Biosynthesis → L-arginine Biosynthesis Superpathways
L-arginine biosynthesis II (acetyl cycle)	Decreased	ARGSYNBSUB-PWY	Biosynthesis → Amino Acid Biosynthesis → Proteinogenic Amino Acid Biosynthesis → L-arginine Biosynthesis
chorismate biosynthesis I	Enriched	ARO-PWY	Biosynthesis → Aromatic Compound Biosynthesis → Chorismate Biosynthesis
pyruvate fermentation to butanoate	Decreased	CENTFERM-PWY	Generation of Precursor Metabolites and Energy → Fermentation → Fermentation of Pyruvate<br>Generation of Precursor Metabolites and Energy → Fermentation → Fermentation to Short-Chain Fatty Acids → Fermentation to Butanoate
3,8-divinyl-chlorophyllide a biosynthesis I (aerobic, light-dependent)	Enriched	CHLOROPHYLL-SYN	Biosynthesis → Tetrapyrrole Biosynthesis → Porphyrin Compound Biosynthesis → Chlorophyll Biosynthesis → Chlorophyllide a Biosynthesis
coenzyme A biosynthesis I (prokaryotic)	Enriched	COA-PWY	Biosynthesis → Cofactor, Carrier, and Vitamin Biosynthesis → Carrier Biosynthesis → Coenzyme A Biosynthesis
superpathway of adenosylcobalamin salvage from cobinamide I	Decreased	COBALSYN-PWY	Biosynthesis → Cofactor, Carrier, and Vitamin Biosynthesis → Enzyme Cofactor Biosynthesis → Cobamide Biosynthesis → Cobinamide Salvage → Adenosylcobalamin Salvage from Cobinamide
superpathway of aromatic amino acid biosynthesis	Enriched	COMPLETE-ARO-PWY	Biosynthesis → Amino Acid Biosynthesis
L-lysine biosynthesis I	Enriched	DAPLYSINESYN-PWY	Biosynthesis → Amino Acid Biosynthesis → Proteinogenic Amino Acid Biosynthesis → L-lysine Biosynthesis
dTDP-L-rhamnose biosynthesis	Decreased	DTDPRHAMSYN-PWY	Biosynthesis → Carbohydrate Biosynthesis → Sugar Biosynthesis → Sugar Nucleotide Biosynthesis → dTDP-sugar Biosynthesis
superpathway of fatty acid biosynthesis initiation (E. coli)	Decreased	FASYN-INITIAL-PWY	Biosynthesis → Fatty Acid and Lipid Biosynthesis → Fatty Acid Biosynthesis
fucose degradation	Decreased	FUCCAT-PWY	Degradation/Utilization/Assimilation → Carbohydrate Degradation → Sugar Degradation
D-galactarate degradation I	Decreased	GALACTARDEG-PWY	Degradation/Utilization/Assimilation → Carboxylate Degradation → Sugar Acid Degradation → D-Galactarate Degradation<br>Degradation/Utilization/Assimilation → Secondary Metabolite Degradation → Sugar Derivative Degradation → Sugar Acid Degradation → D-Galactarate Degradation

D-galacturonate degradation I	Decreased	GALACTUROCAT-PWY	Degradation/Utilization/Assimilation → Carboxylate Degradation → Sugar Acid Degradation → D-Galacturonate Degradation<br>Degradation/Utilization/Assimilation → Secondary Metabolite Degradation → Sugar Derivative Degradation → Sugar Acid Degradation → D-Galacturonate Degradation
superpathway of D-glucarate and D-galactarate degradation	Decreased	GLUCARDEG-PWY	Degradation/Utilization/Assimilation → Carboxylate Degradation → Sugar Acid Degradation → D-Glucarate Degradation<br>Degradation/Utilization/Assimilation → Secondary Metabolite Degradation → Sugar Derivative Degradation → Sugar Acid Degradation → D-Glucarate Degradation
superpathway of D-glucarate and D-galactarate degradation	Decreased	GLUCARGALACTSUPER-PWY	D-galactarate degradation I, *D*-glucarate degradation I
glycogen degradation I	Decreased	GLYCOCAT-PWY	Degradation/Utilization/Assimilation → Carbohydrate Degradation → Polysaccharide Degradation → Glycan Degradation<br>Degradation/Utilization/Assimilation → Carbohydrate Degradation → Polysaccharide Degradation → Glycogen Degradation<br>Degradation/Utilization/Assimilation → Polymeric Compound Degradation → Polysaccharide Degradation → Glycan Degradation<br>Degradation/Utilization/Assimilation → Polymeric Compound Degradation → Polysaccharide Degradation → Glycogen Degradation<br>Glycan Pathways → Glycan Degradation
superpathway of glycol metabolism and degradation	Decreased	GLYCOL-GLYOXDEG-PWY	Degradation/Utilization/Assimilation → Alcohol Degradation
superpathway of glycolysis and the Entner-Doudoroff pathway	Enriched	GLYCOLYSIS-E-D	Generation of Precursor Metabolite and Energy
glyoxylate cycle	Decreased	GLYOXYLATE-BYPASS	Generation of Precursor Metabolite and Energy
heme b biosynthesis II (oxygen-independent)	Enriched	HEMESYN2-PWY	Biosynthesis → Cofactor, Carrier, and Vitamin Biosynthesis → Enzyme Cofactor Biosynthesis → Heme Biosynthesis → Heme b Biosynthesis<br>Biosynthesis → Tetrapyrrole Biosynthesis → Porphyrin Compound Biosynthesis → Heme Biosynthesis → Heme b Biosynthesis
L-histidine biosynthesis	Decreased	HISTSYN-PWY	Biosynthesis → Amino Acid Biosynthesis → Proteinogenic Amino Acid Biosynthesis → L-histidine Biosynthesis
L-methionine biosynthesis III	Decreased	HSERMETANA-PWY	Biosynthesis → Amino Acid Biosynthesis → Proteinogenic Amino Acid Biosynthesis → L-methionine Biosynthesis → L-methionine De Novo Biosynthesis
NAD salvage pathway III (to nicotinamide riboside)	Decreased	NAD-BIOSYNTHESIS-II	Biosynthesis → Cofactor, Carrier, and Vitamin Biosynthesis → Carrier Biosynthesis → Electron Carrier Biosynthesis → NAD Metabolism → NAD Biosynthesis<br>Biosynthesis → Cofactor, Carrier, and Vitamin Biosynthesis → Enzyme Cofactor Biosynthesis → NAD Biosynthesis<br>Biosynthesis → Cofactor, Carrier, and Vitamin Biosynthesis → Enzyme Cofactor Biosynthesis → NAD Metabolism → NAD Biosynthesis
superpathway of L-arginine and L-ornithine degradation	Decreased	ORNARGDEG-PWY	Degradation/Utilization/Assimilation → Amino Acid Degradation → Proteinogenic Amino Acid Degradation → L-arginine Degradation

pentose phosphate pathway	Enriched	PENTOSE-P-PWY	Generation of Precursor Metabolite and Energy → Pentose Phosphate Pathways
peptidoglycan biosynthesis I (meso-diaminopimelate containing)	Enriched	PEPTIDOGLYCANSYN-PWY	Biosynthesis → Cell Structure Biosynthesis → Cell Wall Biosynthesis → Peptidoglycan Biosynthesis
superpathway of phospholipid biosynthesis I (bacteria)	Enriched	PHOSLIPSYN-PWY	Biosynthesis → Fatty Acid and Lipid Biosynthesis → Phospholipid Biosynthesis
superpathway of polyamine biosynthesis II	Decreased	POLYAMINSYN3-PWY	Biosynthesis → Amine and Polyamine Biosynthesis
polyisoprenoid biosynthesis (E. coli)	Enriched	POLYISOPRENSYN-PWY	Biosynthesis → Polyprenyl Biosynthesis → All-trans Polyprenyl Biosynthesis
superpathway of L-isoleucine biosynthesis I	Enriched	PWY-3001	Biosynthesis → Amino Acid Biosynthesis → Proteinogenic Amino Acid Biosynthesis → L-isoleucine Biosynthesis
urea cycle	Enriched	PWY-4984	Degradation/Utilization/Assimilation → Inorganic Nutrient Metabolism → Nitrogen Compound Metabolism
toluene degradation IV (aerobic) (via catechol)	Decreased	PWY-5178	Degradation/Utilization/Assimilation → Aromatic Compound Degradation → Toluene Degradation
toluene degradation III (aerobic) (via p-cresol)	Decreased	PWY-5181	Degradation/Utilization/Assimilation → Aromatic Compound Degradation → Toluene Degradation
Chimeric Pathway: superpathway of aerobic toluene degradation	Decreased	PWY-5183	Degradation/Utilization/Assimilation → Aromatic Compound Degradation → Toluene Degradation
tetrapyrrole biosynthesis I (from glutamate)	Enriched	PWY-5188	Biosynthesis → Tetrapyrrole Biosynthesis
tetrapyrrole biosynthesis II (from glycine)	Enriched	PWY-5189	Biosynthesis → Tetrapyrrole Biosynthesis
meta cleavage pathway of aromatic compounds	Decreased	PWY-5430	Degradation/Utilization/Assimilation → Aromatic Compound Degradation → Benzoate Degradation
adenosylcobalamin biosynthesis from adenosylcobinamide-GDP I	Decreased	PWY-5509	Biosynthesis → Cofactor, Carrier, and Vitamin Biosynthesis → Enzyme Cofactor Biosynthesis → Cobamide Biosynthesis → Cobamide de novo Biosynthesis → Adenosylcobamide Biosynthesis
3,8-divinyl-chlorophyllide a biosynthesis II (anaerobic)	Enriched	PWY-5531	Biosynthesis → Tetrapyrrole Biosynthesis → Porphyrin Compound Biosynthesis → Chlorophyll Biosynthesis → Chlorophyllide a Biosynthesis
CDP-diacylglycerol biosynthesis I	Enriched	PWY-5667	Biosynthesis → Fatty Acid and Lipid Biosynthesis → Phospholipid Biosynthesis → CDP-diacylglycerol Biosynthesis
UMP biosynthesis I	Enriched	PWY-5686	Biosynthesis → Nucleoside and Nucleotide Biosynthesis → Pyrimidine Nucleotide Biosynthesis → Pyrimidine Nucleotide De Novo Biosynthesis → Pyrimidine Ribonucleotide De Novo Biosynthesis → UMP Biosynthesis
allantoin degradation to glyoxylate III	Decreased	PWY-5705	Degradation/Utilization/Assimilation → Amine and Polyamine Degradation → Allantoin Degradation
superpathway of heme b biosynthesis from glutamate	Enriched	PWY-5918	Biosynthesis → Cofactor, Carrier, and Vitamin Biosynthesis → Enzyme Cofactor Biosynthesis → Heme Biosynthesis → Heme b Biosynthesis<br>Biosynthesis → Tetrapyrrole Biosynthesis → Porphyrin Compound Biosynthesis → Heme Biosynthesis → Heme b Biosynthesis

glycogen degradation II	Decreased	PWY-5941	Degradation/Utilization/Assimilation → Carbohydrate Degradation → Polysaccharide Degradation → Glycan Degradation<br>Degradation/Utilization/Assimilation → Carbohydrate Degradation → Polysaccharide Degradation → Glycogen Degradation<br>Degradation/Utilization/Assimilation → Polymeric Compound Degradation → Polysaccharide Degradation → Glycan Degradation<br>Degradation/Utilization/Assimilation → Polymeric Compound Degradation → Polysaccharide Degradation → Glycogen Degradation<br>Glycan Pathways → Glycan Degradation
palmitate biosynthesis II (bacteria and plant cytoplasm)	Decreased	PWY-5971	Biosynthesis → Fatty Acid and Lipid Biosynthesis → Fatty Acid Biosynthesis → Palmitate Biosynthesis
stearate biosynthesis II (bacteria and plants)	Decreased	PWY-5989	Biosynthesis → Fatty Acid and Lipid Biosynthesis → Fatty Acid Biosynthesis → Stearate Biosynthesis
superpathway of guanosine nucleotides de novo biosynthesis II	Enriched	PWY-6125	Biosynthesis → Nucleoside and Nucleotide Biosynthesis → Purine Nucleotide Biosynthesis → Purine Nucleotide De Novo Biosynthesis
6-hydroxymethyl-dihydropterin diphosphate biosynthesis I	Enriched	PWY-6147	Biosynthesis → Cofactor, Prosthetic Group, Electron Carrier, and Vitamin Biosynthesis → Vitamin Biosynthesis → Folate Biosynthesis → 6-Hydroxymethyl-Dihydropterin Diphosphate Biosynthesis
chorismate biosynthesis from 3-dehydroquinate	Enriched	PWY-6163	Biosynthesis → Aromatic Compound Biosynthesis → Chorismate Biosynthesis
superpathway of menaquinol-8 biosynthesis II	Enriched	PWY-6263	Biosynthesis → Cofactor, Prosthetic Group, Electron Carrier, and Vitamin Biosynthesis → Quinol and Quinone Biosynthesis → Menaquinol Biosynthesis
superpathway of adenosylcobalamin salvage from cobinamide II	Decreased	PWY-6269	Biosynthesis → Cofactor, Prosthetic Group, Electron Carrier, and Vitamin Biosynthesis → Vitamin Biosynthesis → Cobamide Biosynthesis → Cobinamide Salvage → Adenosylcobalamin Salvage from Cobinamide
palmitoleate biosynthesis I (from (5Z)-dodec-5-enoate)	Decreased	PWY-6282	Biosynthesis → Fatty Acid and Lipid Biosynthesis → Fatty Acid Biosynthesis → Unsaturated Fatty Acid Biosynthesis → Palmitoleate Biosynthesis
peptidoglycan biosynthesis III (mycobacteria)	Enriched	PWY-6385	Biosynthesis → Cell Structure Biosynthesis → Cell Wall Biosynthesis → Peptidoglycan Biosynthesis
UDP-N-acetylmuramoyl-pentapeptide biosynthesis II (lysine-containing)	Enriched	PWY-6386	Biosynthesis → Cell Structure Biosynthesis → Cell Wall Biosynthesis → UDP-N-Acetylmuramoyl-Pentapeptide Biosynthesis
UDP-N-acetylmuramoyl-pentapeptide biosynthesis I (meso-diaminopimelate containing)	Enriched	PWY-6387	Biosynthesis → Cell Structure Biosynthesis → Cell Wall Biosynthesis → UDP-N-Acetylmuramoyl-Pentapeptide Biosynthesis
pyrimidine deoxyribonucleotides de novo biosynthesis III	Enriched	PWY-6545	Biosynthesis → Nucleoside and Nucleotide Biosynthesis → 2'-Deoxyribonucleotide Biosynthesis → Pyrimidine Deoxyribonucleotide De Novo Biosynthesis<br>Biosynthesis → Nucleoside and Nucleotide Biosynthesis → Pyrimidine Nucleotide Biosynthesis → Pyrimidine Nucleotide De Novo Biosynthesis → Pyrimidine Deoxyribonucleotide De Novo Biosynthesis
norspermidine biosynthesis	Decreased	PWY-6562	Biosynthesis → Amine and Polyamine Biosynthesis

superpathway of Clostridium acetobutylicum acidogenic fermentation	Decreased	PWY-6590	Generation of Precursor Metabolites and Energy → Fermentation → Fermentation of Pyruvate
queuosine biosynthesis I (de novo)	Enriched	PWY-6700	Macromolecule Modification → Nucleic Acid Processing → Queuosine Biosynthesis and Salvage
preQ₀ biosynthesis	Enriched	PWY-6703	Biosynthesis → Secondary Metabolite Biosynthesis
isopropanol biosynthesis (engineered)	Decreased	PWY-6876	Generation of Precursor Metabolites and Energy
thiazole biosynthesis II (aerobic bacteria)	Decreased	PWY-6891	Biosynthesis → Cofactor, Prosthetic Group, Electron Carrier, and Vitamin Biosynthesis → Vitamin Biosynthesis → Thiamine Biosynthesis → Thiazole Biosynthesis
thiazole biosynthesis I (facultative anaerobic bacteria)	Decreased	PWY-6892	Biosynthesis → Cofactor, Prosthetic Group, Electron Carrier, and Vitamin Biosynthesis → Vitamin Biosynthesis → Thiamine Biosynthesis → Thiazole Biosynthesis
superpathway of thiamine diphosphate biosynthesis II	Decreased	PWY-6895	Biosynthesis → Cofactor, Prosthetic Group, Electron Carrier, and Vitamin Biosynthesis → Vitamin Biosynthesis → Thiamine Biosynthesis
(S)-propane-1,2-diol degradation	Decreased	PWY-7013	Degradation/Utilization/Assimilation → Alcohol Degradation<br>Generation of Precursor Metabolite and Energy → Fermentation → Fermentation to Short-Chain Fatty Acids → Fermentation to Propanoate
3,8-divinyl-chlorophyllide <i>a</i> biosynthesis III (aerobic, light independent)	Enriched	PWY-7159	Biosynthesis → Cofactor, Prosthetic Group, Electron Carrier, and Vitamin Biosynthesis → Porphyrin Compound Biosynthesis → Chlorophyll Biosynthesis → Chlorophyllide a Biosynthesis
pyrimidine deoxyribonucleotide phosphorylation	Enriched	PWY-7197	Biosynthesis → Nucleoside and Nucleotide Biosynthesis → Pyrimidine Nucleotide Biosynthesis → Pyrimidine Nucleotide Salvage
pyrimidine deoxyribonucleosides salvage	Decreased	PWY-7199	Biosynthesis → Nucleoside and Nucleotide Biosynthesis → Pyrimidine Nucleotide Biosynthesis → Pyrimidine Nucleotide Salvage
superpathway of pyrimidine deoxyribonucleotides de novo biosynthesis	Enriched	PWY-7211	Biosynthesis → Nucleoside and Nucleotide Biosynthesis → 2'-Deoxyribonucleotide Biosynthesis → Pyrimidine Deoxyribonucleotide De Novo Biosynthesis<br>Biosynthesis → Nucleoside and Nucleotide Biosynthesis → Pyrimidine Nucleotide Biosynthesis → Pyrimidine Nucleotide De Novo Biosynthesis → Pyrimidine Deoxyribonucleotide De Novo Biosynthesis
adenosine ribonucleotides de novo biosynthesis	Enriched	PWY-7219	Biosynthesis → Nucleoside and Nucleotide Biosynthesis → Purine Nucleotide Biosynthesis → Purine Nucleotide De Novo Biosynthesis → Purine Riboucleotide De Novo Biosynthesis
guanosine ribonucleotides de novo biosynthesis	Enriched	PWY-7221	Biosynthesis → Nucleoside and Nucleotide Biosynthesis → Purine Nucleotide Biosynthesis → Purine Nucleotide De Novo Biosynthesis → Purine Riboucleotide De Novo Biosynthesis
superpathway of guanosine nucleotides de novo biosynthesis I	Enriched	PWY-7228	Biosynthesis → Nucleoside and Nucleotide Biosynthesis → Purine Nucleotide Biosynthesis → Purine Nucleotide De Novo Biosynthesis
superpathway of adenosine nucleotides de novo biosynthesis I	Enriched	PWY-7229	Biosynthesis → Nucleoside and Nucleotide Biosynthesis → Purine Nucleotide Biosynthesis → Purine Nucleotide De Novo Biosynthesis
TCA cycle VII (acetate-producers)	Enriched	PWY-7254	Generation of Precursor Metabolite and Energy → TCA cycle

superpathway of UDP-N-acetylglucosamine-derived O-antigen building blocks biosynthesis	Decreased	PWY-7332	Biosynthesis → Carbohydrate Biosynthesis → Sugar Biosynthesis → Sugar Nucleotide Biosynthesis → UDP-sugar Biosynthesis
1,4-dihydroxy-6-naphthoate biosynthesis II	Enriched	PWY-7371	Biosynthesis → Cofactor, Prosthetic Group, Electron Carrier, and Vitamin Biosynthesis → Quinol and Quinone Biosynthesis → 1,4-dihydroxy-6-naphthoate biosynthesis
superpathway of demethylmenaquinol-6 biosynthesis II	Enriched	PWY-7373	Biosynthesis → Cofactor, Prosthetic Group, Electron Carrier, and Vitamin Biosynthesis → Quinol and Quinone Biosynthesis → Demethylmenaquinol Biosynthesis → Demethylmenaquinol-6 Biosynthesis
1,4-dihydroxy-6-naphthoate biosynthesis I	Enriched	PWY-7374	Biosynthesis → Cofactor, Prosthetic Group, Electron Carrier, and Vitamin Biosynthesis → Quinol and Quinone Biosynthesis → 1,4-dihydroxy-6-naphthoate biosynthesis
L-arginine biosynthesis IV (archaebacteria)	Decreased	PWY-7400	Biosynthesis → Amino Acid Biosynthesis → Proteinogenic Amino Acid Biosynthesis → L-arginine Biosynthesis
6-hydroxymethyl-dihydropterin diphosphate biosynthesis III (Chlamydia)	Enriched	PWY-7539	Biosynthesis → Cofactor, Prosthetic Group, Electron Carrier, and Vitamin Biosynthesis → Vitamin Biosynthesis → Folate Biosynthesis → 6-Hydroxymethyl-Dihydropterin Diphosphate Biosynthesis
oleate biosynthesis IV (anaerobic)	Decreased	PWY-7664	Biosynthesis → Fatty Acid and Lipid Biosynthesis → Fatty Acid Biosynthesis → Unsaturated Fatty Acid Biosynthesis → Oleate Biosynthesis
ADP-L-glycero-beta-D-manno-heptose biosynthesis	Enriched	PWY0-1241	Biosynthesis → Carbohydrate Biosynthesis → Sugar Biosynthesis → Sugar Nucleotide Biosynthesis → ADP-sugar Biosynthesis
purine ribonucleosides degradation	Enriched	PWY0-1296	Degradation/Utilization/Assimilation → Nucleoside and Nucleotide Degradation → Purine Nucleotide Degradation
CDP-diacylglycerol biosynthesis II	Enriched	PWY0-1319	Biosynthesis → Fatty Acid and Lipid Biosynthesis → Phospholipid Biosynthesis → CDP-diacylglycerol Biosynthesis
methylphosphonate degradation I	Decreased	PWY0-1533	Degradation/Utilization/Assimilation → Inorganic Nutrient Metabolism → Phosphorus Compound Metabolism → Methylphosphonate Degradation
superpathway of pyrimidine ribonucleotides de novo biosynthesis	Enriched	PWY0-162	Biosynthesis → Nucleoside and Nucleotide Biosynthesis → Pyrimidine Nucleotide Biosynthesis → Pyrimidine Nucleotide De Novo Biosynthesis → Pyrimidine Ribonucleotide De Novo Biosynthesis
allantoin degradation IV (anaerobic)	Decreased	PWY0-41	Degradation/Utilization/Assimilation → Amine and Polyamine Degradation → Allantoin Degradation
superpathway of pyridoxal 5'-phosphate biosynthesis and salvage	Decreased	PWY0-845	Biosynthesis → Cofactor, Carrier, and Vitamin Biosynthesis → Enzyme Cofactor Biosynthesis → Vitamin B6 Biosynthesis<br>Biosynthesis → Cofactor, Carrier, and Vitamin Biosynthesis → Vitamin Biosynthesis → Vitamin B6 Biosynthesis
(5Z)-dodecenoate biosynthesis I	Decreased	PWY0-862	Biosynthesis → Fatty Acid and Lipid Biosynthesis → Fatty Acid Biosynthesis → Unsaturated Fatty Acid Biosynthesis → (5Z)-dodecenoate Biosynthesis
mycothiol biosynthesis	Enriched	PWY1G-0	Biosynthesis → Cofactor, Prosthetic Group, Electron Carrier, and Vitamin Biosynthesis → Reductant Biosynthesis

nitrate reduction VI (assimilatory)	Decreased	PWY490-3	Degradation/Utilization/Assimilation → Inorganic Nutrient Metabolism → Nitrogen Compound Metabolism → Nitrate Reduction
phosphatidylglycerol biosynthesis I (plastidic)	Enriched	PWY4FS-7	Biosynthesis → Fatty Acid and Lipid Biosynthesis → Phospholipid Biosynthesis → Phosphatidylglycerol Biosynthesis
phosphatidylglycerol biosynthesis II (non-plastidic)	Enriched	PWY4FS-8	Biosynthesis → Fatty Acid and Lipid Biosynthesis → Phospholipid Biosynthesis → Phosphatidylglycerol Biosynthesis
mycolate biosynthesis	Decreased	PWYG-321	Biosynthesis → Fatty Acid and Lipid Biosynthesis → Fatty Acid Biosynthesis
TCA cycle VI (Helicobacter)	Enriched	REDCITCYC	Generation of Precursor Metabolite and Energy → TCA cycle
flavin biosynthesis I (bacteria and plants)	Enriched	RIBOSYN2-PWY	Bacteria <bacteria>, Viridiplantae
superpathway of L-serine and glycine biosynthesis I	Enriched	SER-GLYSYN-PWY	Biosynthesis → Amino Acid Biosynthesis
superpathway of thiamine diphosphate biosynthesis I	Decreased	THISYN-PWY	Biosynthesis → Cofactor, Prosthetic Group, Electron Carrier, and Vitamin Biosynthesis → Vitamin Biosynthesis → Thiamine Biosynthesis
superpathway of L-threonine biosynthesis	Enriched	THRESYN-PWY	Biosynthesis → Amino Acid Biosynthesis → Proteinogenic Amino Acid Biosynthesis → L-threonine Biosynthesis
L-tryptophan biosynthesis	Enriched	TRPSYN-PWY	Biosynthesis → Amino Acid Biosynthesis → Proteinogenic Amino Acid Biosynthesis → L-tryptophan Biosynthesis
UDP-N-acetyl-D-glucosamine biosynthesis I	Enriched	UDPNAGSYN-PWY	Biosynthesis → Carbohydrate Biosynthesis → Sugar Biosynthesis → Sugar Nucleotide Biosynthesis → UDP-sugar Biosynthesis → UDP-N-acetyl-D-glucosamine Biosynthesis

Supplementary Table 2: Percentage of change of the variables throughout the follow-up.

	Non-PPIs users	PPIs users	*H. pylori*
Weight change 3 months	-21.81 ± 3.67	-17.84 ± 2.42*	-19.55 ± 4.63
Weight change 6 months	-29.98 ± 5.03	-26.44 ± 6.62	-25.69 ± 5.65
Weight change 1 year	-38.96 ± 7.48	-31.44 ± 5.19	-28.38 ± 5.63#
BMI change 3 months	-21.81 ± 3.66	-17.89 ± 2.70*	-19.54 ± 4.64
BMI change 6 months	-29.98 ± 5.02	-26.35 ± 6.74	-25.70 ± 5.64
BMI change 1 year	-38.95 ± 7.49	-31.47 ± 5.22	-28.39 ± 5.63#
Glucose change 3 months	-15.00 ± 11.08	-10.79 ± 17.34	-15.31 ± 17.26
Glucose change 6 months	-15.27 ± 16.98	-17.90 ± 12.01	-15.42 ± 20.21
Glucose change 1 year	-20.22 ± 13.39	-18.39 ± 16.18	-21.82 ± 19.82
Insulin change 3 months	-51.92 ± 39.31	-59.11 ± 19.18	-42.84 ± 32.04
Insulin change 6 months	-49.98 ± 52.97	-54.13 ± 26.94	-53.15 ± 24.47
Insulin change 1 year	-69.96 ± 22.93	-63.96 ± 26.70	-56.61 ± 19.44
HOMA-IR change 3 months	-57.10 ± 40.12	-61.29 ± 23.84	-49.47 ± 33.07
HOMA-IR change 6 months	-50.15 ± 62.82	-60.63 ± 25.93	-57.49 ± 22.71
HOMA-IR change 1 year	-77.04 ± 19.89	-67.26 ± 31.83	-58.85 ± 22.91
HbA1c change 3 months	-8.59 ± 6.63	-6.72 ± 5.64	-12.41 ± 11.48
HbA1c change 6 months	-8,92 ± 7.20	-7.20 ± 3.40	-12.42 ± 13.56
HbA1c change 1 year	-8.55 ± 7.30	-7.91 ± 5.13	-12.68 ± 11.59
Cholesterol change 3 months	1.34 ± 13.51	-2.44 ± 12.06	-3.27 ± 10.17
Cholesterol change 6 months	-0.54 ± 14.16	0.57 ± 18.08	3.65 ± 14.77
Cholesterol change 1 year	2.88 ± 19.48	-1.93 ± 18.74	-1.58 ± 10.23
Triglycerides change 3 months	-2.81 ± 30.96	-14.72 ± 25.57	-15.85 ± 41.25
Triglycerides change 6 months	-17.74 ± 28.98	-29.82 ± 18.08	-31.51 ± 38.58
Triglycerides change 1 year	-34.94 ± 17.56	-23.35 ± 14.14	-36.47 ± 35.94
HDL- chol. change 3 months	-5.46 ± 19.91	-1.39 ± 22.05	1.20 ± 8.76
HDL-chol. change 6 months	5.05 ± 26.14	13.82 ± 34.97	9.58 ± 21.95
HDL-chol. change 1 year	19.96 ± 29.66	16.33 ± 27.13	19.31 ± 23.43
LDL- chol. change 3 months	7.35 ± 18.87	3.56 ± 22.78	5.88 ± 22.40
LDL-chol. change 6 months	4.59 ± 17.31	7.79 ± 39.55	19.92 ± 19.16
LDL-chol. change 1 year	7.41 ± 27.68	-4.46 ± 21.28	7.89 ± 23.92

The results are given as the mean ± SD. BMI: Body mass index. HOMA-IR: homeostasis model assessment of insulin resistance index. HbA1c: glycated haemoglobin. Kruskal-Wallis test *$p<0.05$ between non-PPIs users and PPIs users. #$p<0.05$ between non-PPIs users and *H.pylori* group.

Supplementary Figure S1: Pooled gut microbiota composition of all subjects included in the study.

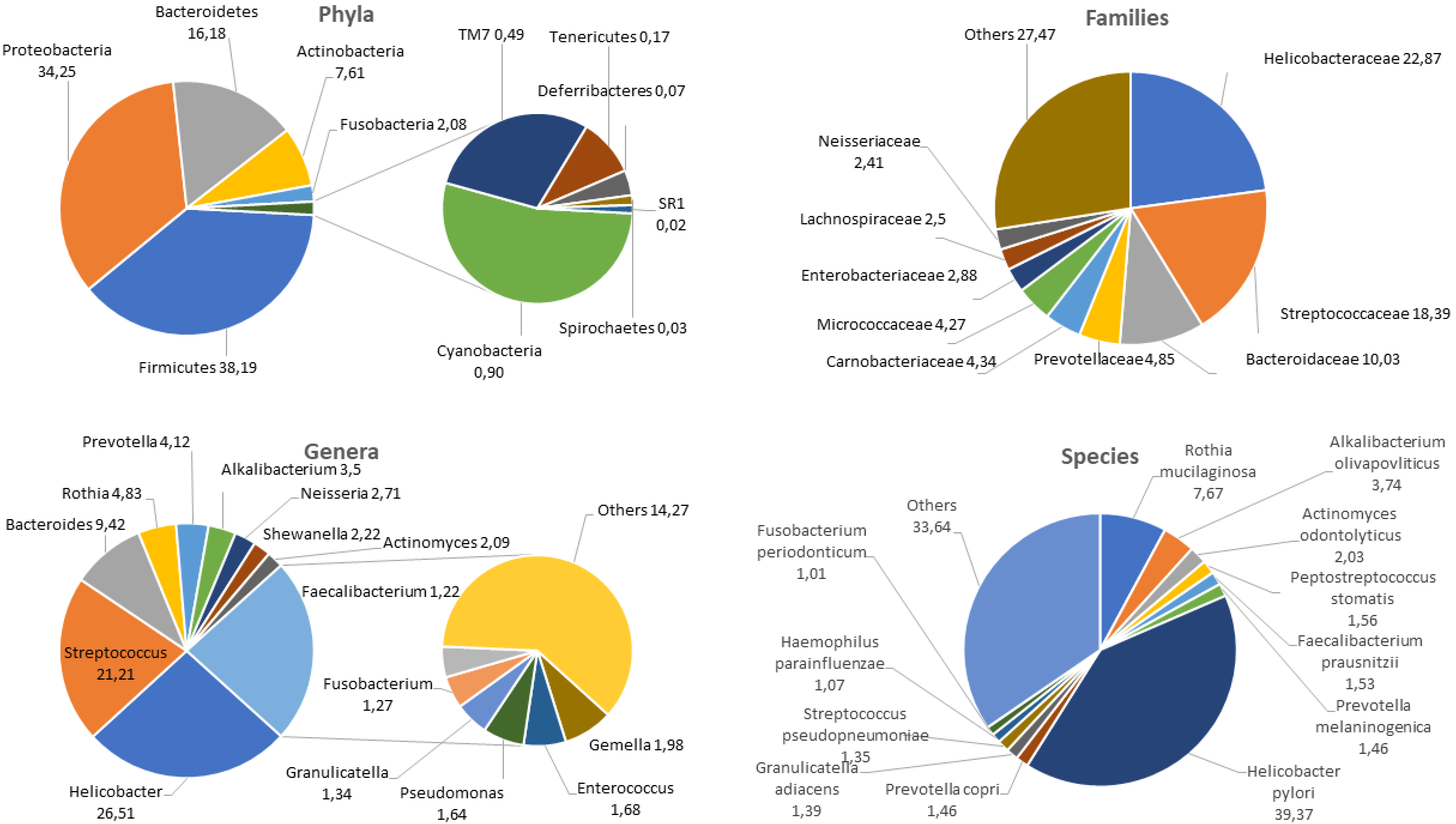
Phyla
Bacteroidetes 16,18
Proteobacteria 34,25
Actinobacteria 7,61
Fusobacteria 2,08
TM7 0,49
Tenericutes 0,17
Deferribacteres 0,07
SR1 0,02
Spirochaetes 0,03
Cyanobacteria 0,90
Firmicutes 38,19
Families
Others 27,47
Helicobacteraceae 22,87
Neisseriaceae 2,41
Lachnospiraceae 2,5
Enterobacteriaceae 2,88
Micrococcaceae 4,27
Carnobacteriaceae 4,34
Prevotellaceae 4,85
Bacteroidaceae 10,03
Streptococcaceae 18,39
Genera
Prevotella 4,12
Alkalibacterium 3,5
Rothia 4,83
Neisseria 2,71
Bacteroides 9,42
Shewanella 2,22
Actinomyces 2,09
Others 14,27
Faecalibacterium 1,22
Streptococcus 21,21
Fusobacterium 1,27
Gemella 1,98
Granulicatella 1,34
Pseudomonas 1,64
Enterococcus 1,68
Helicobacter 26,51
Species
Rothia mucilaginosa 7,67
Alkalibacterium olivapovliticus 3,74
Others 33,64
Actinomyces odontolyticus 2,03
Fusobacterium periodonticum 1,01
Peptostreptococcus stomatis 1,56
Faecalibacterium prausnitzii 1,53
Haemophilus parainfluenzae 1,07
Prevotella melaninogenica 1,46
Streptococcus pseudopneumoniae 1,35
Granulicatella adiacens 1,39
Prevotella copri 1,46
Helicobacter pylori 39,37

Supplementary Figure S2a: Venn diagram of the core microbiomes identified in 100% of studied samples at family level.

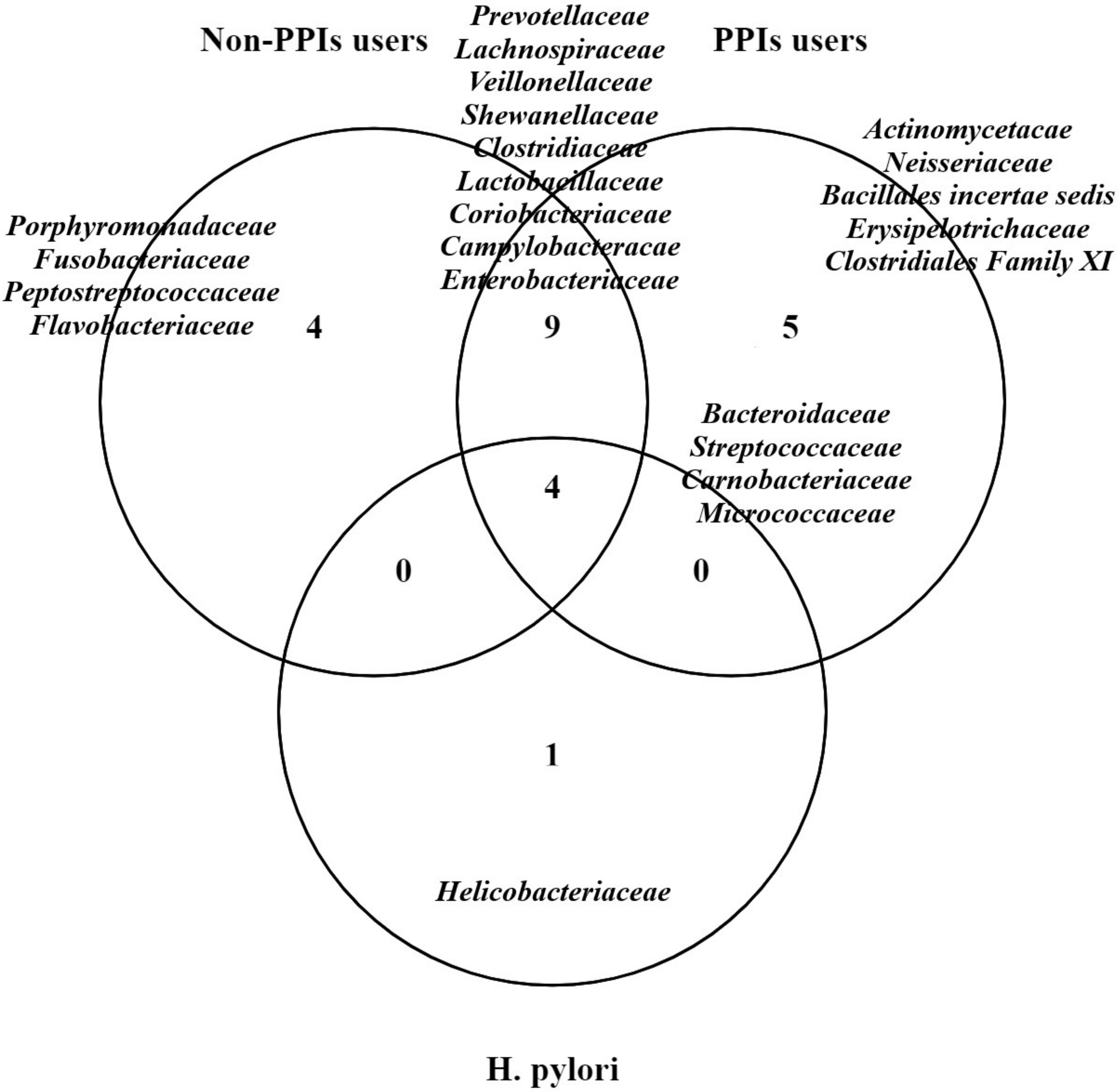
Non-PPIs users
PPIs users
Prevotellaceae
Lachnospiraceae
Veillonellaceae
Shewanellaceae
Clostridiaceae
Lactobacillaceae
Coriobacteriaceae
Campylobacteracae
Enterobacteriaceae
Actinomycetacae
Neisseriaceae
Bacillales incertae sedis
Erysipelotrichaceae
Clostridiales Family XI
Porphyromonadaceae
Fusobacteriaceae
Peptostreptococcaceae
Flavobacteriaceae
4
9
5
Bacteroidaceae
Streptococcaceae
Carnobacteriaceae
Micrococcaceae
4
0
0
1
Helicobacteriaceae
H. pylori

Supplementary Figure S2b: Venn diagram of the core microbiomes identified in 100% of studied samples at genus level.

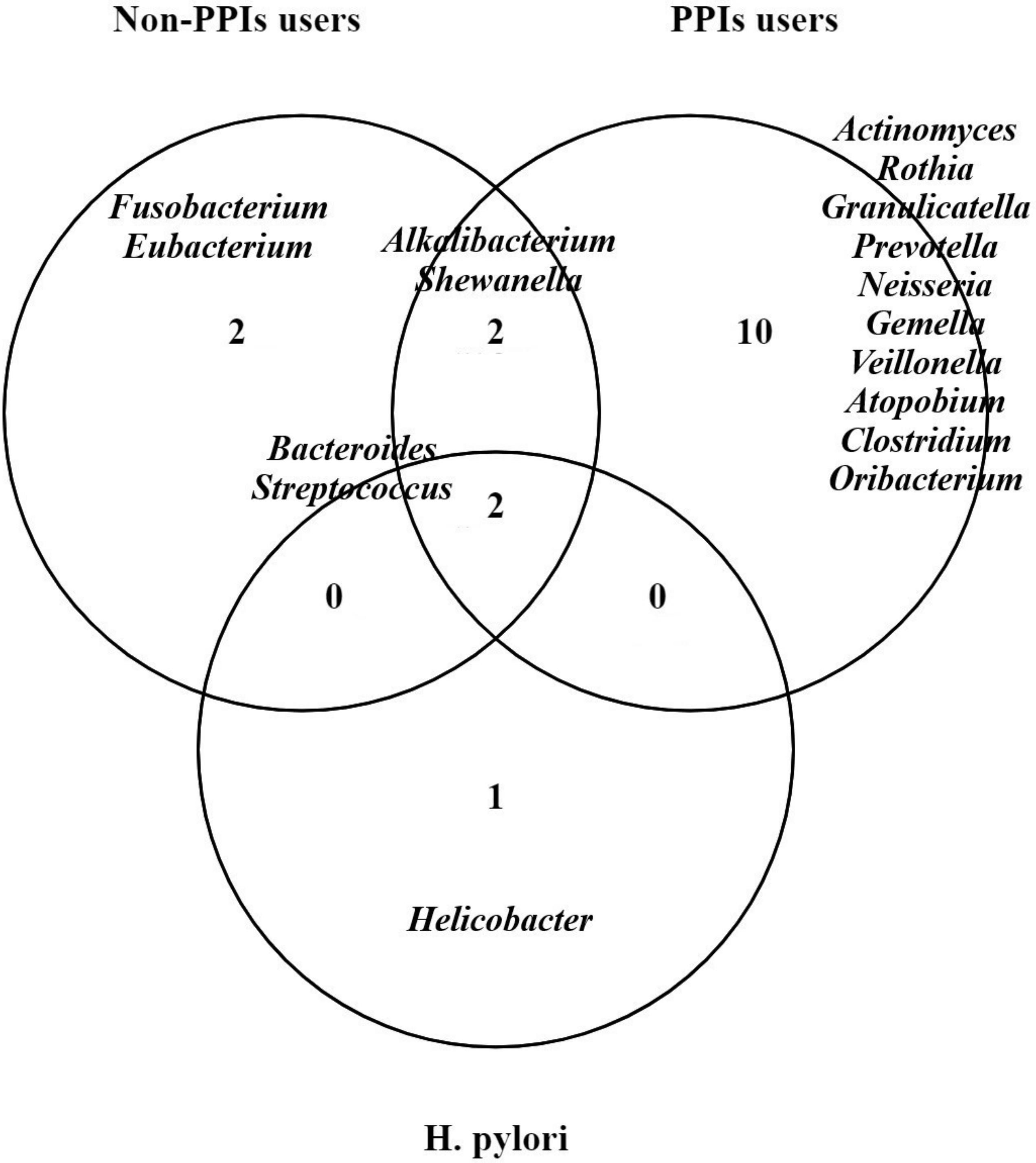

Non-PPIs users
PPIs users
Fusobacterium
Eubacterium
2
Alkalibacterium
Shewanella
2
10
Actinomyces
Rothia
Granulicatella
Prevotella
Neisseria
Gemella
Veillonella
Atopobium
Clostridium
Oribacterium
Bacteroides
Streptococcus
2
0
0
1
Helicobacter
H. pylori

Supplementary Figure S2c: Venn diagram of the core microbiomes identified in 100% of studied samples at species level.

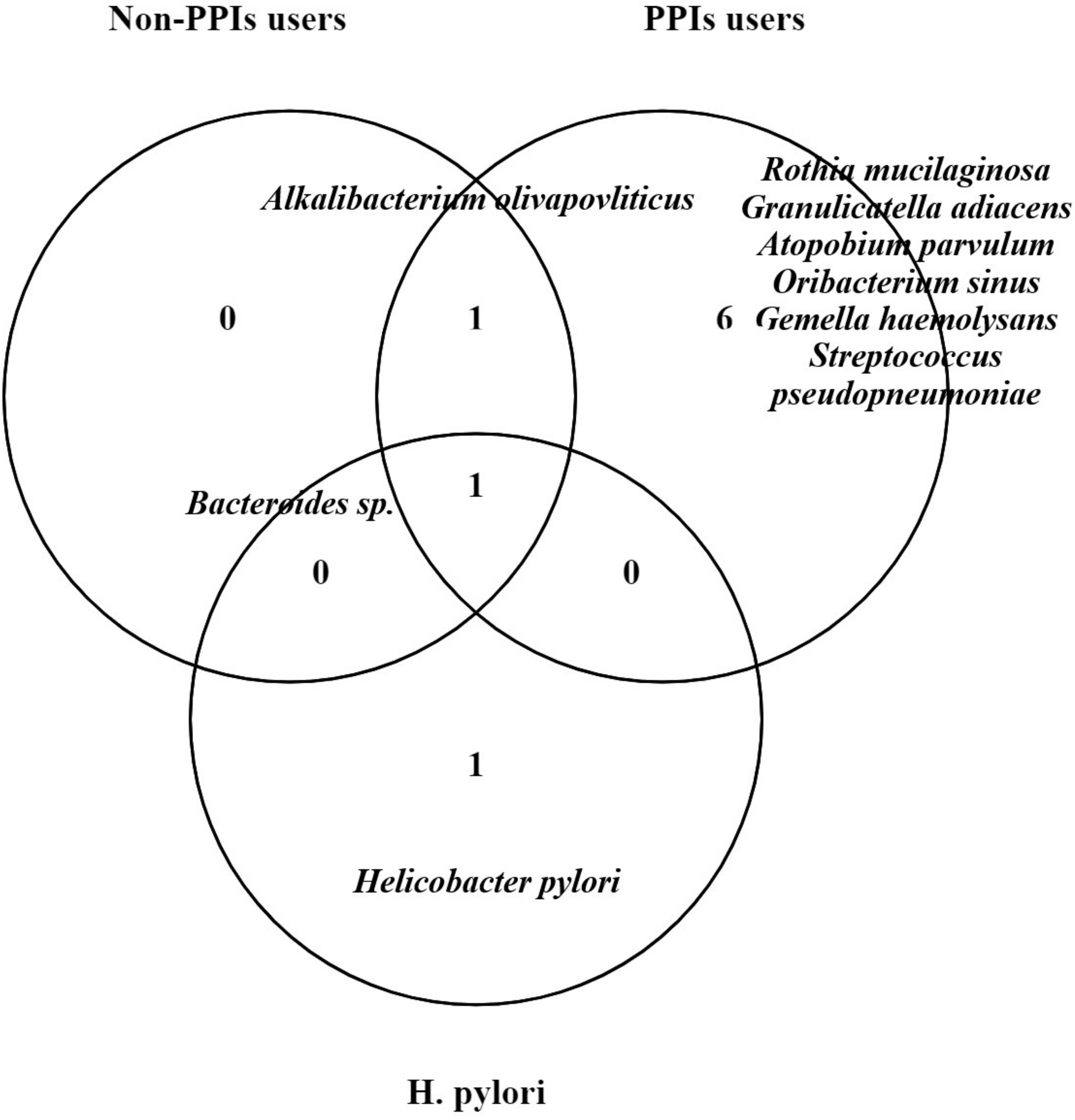
Non-PPIs users
PPIs users
Alkalibacterium olivapovliticus
Rothia mucilaginosa
Granulicatella adiacens
Atopobium parvulum
Oribacterium sinus
Gemella haemolysans
Streptococcus
pseudopneumoniae
0
1
6
Bacteroides sp.
1
0
0
1
Helicobacter pylori
H. pylori

Supplementary Figure S3: Relative abundance of the KEGG pathways significantly different between non-PPIs users (orange bars) and PPIs users (blue bars).

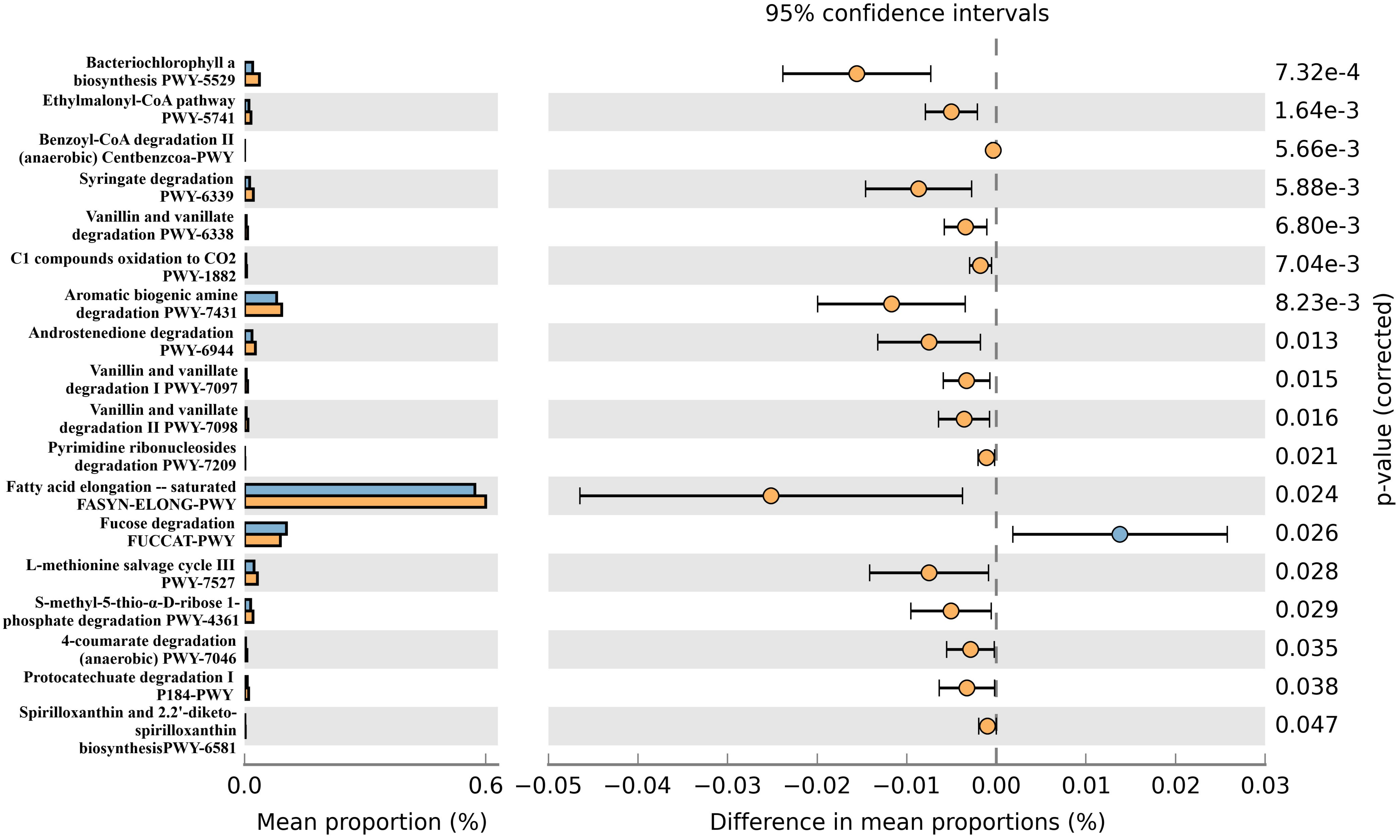

95% confidence intervals
Bacteriochlorophyll a biosynthesis PWY-5529
Ethylmalonyl-CoA pathway PWY-5741
Benzoyl-CoA degradation II (anaerobic) Centbenzcoa-PWY
Syringate degradation PWY-6339
Vanillin and vanillate degradation PWY-6338
C1 compounds oxidation to CO2 PWY-1882
Aromatic biogenic amine degradation PWY-7431
Androstenedione degradation PWY-6944
Vanillin and vanillate degradation I PWY-7097
Vanillin and vanillate degradation II PWY-7098
Pyrimidine ribonucleosides degradation PWY-7209
Fatty acid elongation -- saturated FASYN-ELONG-PWY
Fucose degradation FUCCAT-PWY
L-methionine salvage cycle III PWY-7527
S-methyl-5-thio-α-D-ribose 1-phosphate degradation PWY-4361
4-coumarate degradation (anaerobic) PWY-7046
Protocatechuate degradation I P184-PWY
Spirilloxanthin and 2.2'-diketo-spirilloxanthin biosynthesisPWY-6581
7.32e-4
1.64e-3
5.66e-3
5.88e-3
6.80e-3
7.04e-3
8.23e-3
0.013
0.015
0.016
0.021
0.024
0.026
0.028
0.029
0.035
0.038
0.047
p-value (corrected)
0.0
0.6
Mean proportion (%)
−0.05
−0.04
−0.03
−0.02
−0.01
0.00
0.01
0.02
0.03
Difference in mean proportions (%)
